# Crystallographic Texture Evolution in 3D Printed Polyethylene Reactor Blends

**DOI:** 10.1021/acsomega.4c00387

**Published:** 2024-05-01

**Authors:** Sahitya Movva, Carl G. Schirmeister, Timo Hees, David Tavakoli, Erik H. Licht, Rolf Mülhaupt, Hamid Garmestani, Karl I. Jacob

**Affiliations:** † School of Materials Science and Engineering, Georgia Institute of Technology, Atlanta, Georgia 30332, United States; ‡ Freiburg Materials Research Center FMF and Institute for Macromolecular Chemistry, Albert-Ludwigs-University Freiburg, Stefan-Meier-Str. 21, Freiburg D-79104, Germany; § Basell Sales & Marketing B.V., LyondellBasell Industries, Industriepark Höchst, Frankfurt a.M. D-65926, Germany; ∥ Sustainability Center Freiburg, Ecker-Str. 4, Freiburg D-79104, Germany; ⊥ Intel Corporation, 2501 NE Century Blvd, Hillsboro, Oregon 97124, United States; # G.W. Woodruff School of Mechanical Engineering, Georgia Institute of Technology, Atlanta, Georgia 30332, United States

## Abstract

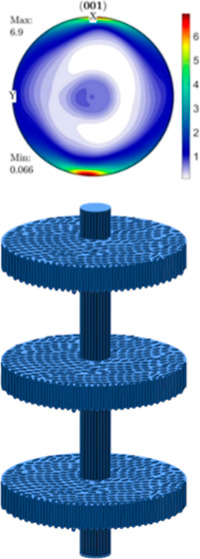

In this work, crystallographic texture evolution in 3D printed trimodal polyethylene (PE) blends and high-density PE (HDPE) benchmark material were investigated to quantify the resulting material anisotropy, and the results were compared to materials made from conventional injection molded (IM) samples. Trimodal PE reactor blends consisting of HDPE, ultrahigh molecular weight PE (UHMWPE), and HDPE_wax have been used for 3D printing and injection molding. Changes in the preferred orientation and distribution of crystallites, i.e., texture evolution, were quantified utilizing the wide angle X-ray diffraction through pole figures and orientation distribution functions (ODFs) for 3D printed and IM samples. Since the change in weight-average molecular weight (*M*_w_) of the blend was expected to significantly affect the resulting crystallinity and orientation, the overall *M*_w_ of the trimodal PE blend was varied while keeping the UHMWPE component weight fraction to 10% in the blend. The resulting texture was analyzed by varying the overall *M*_w_ of the trimodal blend and the process parameters in 3D printing and compared to the texture of conventional IM samples. The printing speed and orientation (defined with respect to the axis along the length of the samples) were used as the variable process parameters for 3D printing. The degree of anisotropy increases with an increase in the nonuniform distribution of intensities in pole figures and ODFs. All the highest intensity major texture components in IM and 3D printed samples (0° printing orientation) of reactor blends are observed to have crystals oriented in [001] or [001̅]. Overall, for the same throughput, 3D printed samples in the 0° orientation showed greater texture evolution and higher anisotropy compared to IM samples. Most notably, an increase in 3D printing speed increased the crystalline distribution closer to the 0° direction, increasing the anisotropy, while deviation from this printing orientation reduced crystalline distribution closer to the 0° direction, thus increasing isotropy. This demonstrates that tailoring material properties in specific directions can be achieved more effectively with 3D printing than with the injection molding process. Change in the overall *M*_w_ of the trimodal PE blend changed the preferential orientation distribution of the crystal planes to some degree. However, the degree of anisotropy remained the same in almost all cases, indicating that the effect of molecular weight distribution is not as significant as the printing speed and printing orientation in tailoring the resulting properties. The 3D printing process parameters (speed and orientation) were shown to have more influence on the texture than the material parameters associated with the blend.

## 1 Introduction

Polyethylene (PE) is a lightweight, versatile, durable, and efficiently recyclable semicrystalline thermoplastic hydrocarbon polymer.^1,2^ It is one of the most widely produced polymers, with tens of millions of tons being produced worldwide each year. It has a number of applications in the packaging, automotive, medical and healthcare, electrical, fibers, and textile industries, along with other high-performance applications. It is also used in making pipes, hoses, and fittings, in agriculture, and in wiring and cables, etc. Most importantly, PE is utilized as a gold-standard biomaterial for surgical implants.^3,4^ Out of all the major PE grades commercially available, high-density PE (HDPE) and ultrahigh molecular weight PE (UHMWPE) with a molar mass *M*_w_ > 10^6^ g mol^–1^ are the most versatile for technical and medical applications and are integral parts of the reactor blends studied in this work.

HDPE is an economical thermoplastic with no or a low degree of branching. It is flexible, weather-resistant, and tough at very low temperatures.^5^ It has excellent resistance to most solvents and excellent insulating properties. It has a higher tensile strength compared to other grades of PE, good low-temperature resistance, and very low water absorption. Moreover, they are Food and Drug Administration-compliant and biocompatible. Some disadvantages of HDPE include susceptibility to stress cracking, lower stiffness than polypropylene, high mold shrinkage, and low heat resistance. However, the excellent combination of properties and the ability to be engineered according to end-use requirements make HDPE an ideal material for diverse applications across various industries.

UHMWPE can be spun into fibers and drawn or stretched into a highly crystalline polymer with high stiffness and tensile strength greater than that of steel, allowing it to be used in bulletproof vests.^6^ It has other excellent mechanical properties, such as high impact strength, outstanding abrasion resistance, and a low coefficient of friction. Thus far, in small concentrations it helps to significantly increase the stress crack resistance of HDPE. UHMWPE is almost inert at moderate temperatures, which allows it to be very useful in corrosive and aggressive environments. Even at high temperatures, it exhibits chemical resistance to several solvents except for aromatic, halogenated hydrocarbons, and strong oxidizing materials such as nitric acid. Due to these exceptional properties, UHMWPE is highly useful in high-performance applications. It is particularly suitable for high-wear applications such as liners and other equipment.

The microstructure of a material strongly depends on how the material is processed, which in turn affects the resultant final properties of the material. PE generally has an orthorhombic crystal structure within a lamella. Lamellar crystal structures can be found distributed within the volume, with their major axis forming some directionality for the crystals. They can also cluster together to form various hierarchical structures, such as bands of folded chain crystals connected together by tie molecules forming spherulitic structures usually under a quiescent crystallization condition. Spherulites are spherically symmetric with radial crystalline lamella separated by amorphous regions and thus do not have any orientation in the unstressed natural state.^7^ PE crystallized from melt under low strain rates generally forms spherulite crystals, while PE crystallized from solution primarily form chain folded single crystals. PE crystallized from melt or solution form shish kebab fibrous crystals with other crystal forms still present in lesser amounts under flow. Shish kebab is a combination structure, with extended chain crystals as the roughly cylindrical core and folded chain crystals on the flange connected to the core cylinder. The lamellar crystals are further oriented under a shear flow in extrusion, giving rise to additional crystalline orientation that can be studied using scattering or diffraction techniques. Depending on the method of processing the polymer undergoes, the size, the number of crystallites present, and their orientation can vary, affecting the resultant structural architecture and properties.^8^ It has been established that when both UHMWPE and HDPE are present together, their molecular chains can affect each other during the structure evolution resulting in improved overall structure of the blends.^9,10^ It has also been reported that UHMWPE chains become more easily disentangled when blended with HDPE in melt processing, and also the HDPE addition enhanced the mobility and crystal structure of UHMWPE significantly.^11−13^ In HDPE/UHMWPE blends, it has been reported that the crystallization of UHMWPE could induce the crystallization of HDPE at higher temperatures resulting in lower crystallization rates of the blends than those of their neat components.^14^ In addition, the crystallite size was found to increase with an increase in the UHMWPE content in the HDPE/UHMWPE blends.^15,16^ HDPE is generally processed by using natural gas or oil cracking followed by a highly energy- and resource-efficient solvent-free catalytic polymerization process. HDPE is exceptionally easy to process from the melt via, e.g., injection molding, extrusion, and additive manufacturing, while UHMWPE—owing to the extremely high viscosity resulting from higher chain entanglements—is usually manufactured from resin using compression molding and ram extrusion.^17−19^ In order to combine the outstanding properties of UHMWPE with the good processability of HDPE, various attempts have been made to melt blend both polymers, which is, however, limited to a few percent of UHMWPE in HDPE.^20^ At the beginning of the 21st century, this limitation was overcome by developing supported multisite catalysts that form nanophase-separated disentangled UHMWPE combined with HDPE by ethylene polymerization in a single reactor. Due to the nanophase separation of unentangled UHMWPE, high UHMWPE contents are tolerated in conventional injection molding when low-molecular-weight HDPE is a lubricant and processing aid.^21,22,24^ During melt processing, such as extrusion or injection molding, the flow-induced coil–stretch transition of the disentangled UHMWPE forms and promotes oriented extended-chain UHMWPE nanostructures in the direction of the flow. These extended-chain UHMWPE nanostructures nucleate, as so-called shish, the epitaxial crystallization of HDPE to yield shish-kebab structures that remain in the solidified material as reinforcing phases, leading to substantially improved stiffness, strength, toughness, and high wear resistance.^24−26^ Comparatively, there are more spherulite crystals in injection molding compared to chain folded crystals while there are more extended chain crystals in extrusion in general The amount, type, and structure of crystals obtained through injection molding depends on parameters involved like barrel temperature, injection speed/time/volume, holding pressure, holding time, and cooling time while those obtained through extrusion depends on parameters like temperature of molten polymer, thickness of film deposited, roller pressure, extrusion speed, and air gap.^27,28^

Understanding the evolution of microstructure and the resultant texture, defined as the preferred alignment of crystallographic orientations in a polycrystalline medium,^7^ has been of great interest among the materials community.^8,29−37,37^ Additive manufacturing techniques, especially fused filament fabrication (FFF), also known as fused deposition modeling (FDM), an extrusion-based 3D printing technique, has shown to be very promising to fabricate sustainable customized complex materials.^38,39^ 3D printing is more customizable and less expensive compared to injection molding. It is also possible to have better control of the process through its parameters like 3D printing speed, orientation, and temperature, thereby obtaining better control of microstructure and hence the resultant properties.^23^

There are numerous studies involving the microstructure of HDPE and UHMWPE and their blends processed through different ways,^39−51^ as well as X-ray diffraction studies of HDPE or UHMWPE-containing polymers.^15,36,52−60^ There have been studies on the evolution of microstructures in UHMWPE/HDPE blend fibers prepared by melt spinning,^15^ control of subinclusion microstructure in ternary blends consisting of HDPE,^43^ microstructural characterization of HDPE/polyamide blend through selective laser sintering processing,^44^ microstructural evaluation of HDPE along with its other properties after weathering,^40^ assessment of microstructure of HDPE post chemical recycling,^41^ post reinforcement for use as shielding materials against nuclear radiation,^42^ effect of various reinforcements on microstructure of UHMWPE consisting composites,^46,47^ studies on microstructure of modified UHMWPE^50^ and UHMWPE consisting blend foams,^51^ and many more. Pandit et al. investigated carbon-fiber-reinforced HDPE composites to improve printability of HDPE using FDM which otherwise show warping, shrinking, and weak bonding between HDPE and substrate.^39^ There have also been studies that confirm the presence of shish kebab structures of UHMWPE fibers prepared by gel spinning^61−63^ and the formation of shish-kebab structure of UHMWPE during injection molding of UHMWPE/HDPE blends as well as effect of change in UHMWPE molecular weight when melt blended with HDPE.^48^ Our previous work investigated crystallographic orientation or texture evolution in UHMWPE during uniaxial tension.^8^ For polymer products, texture plays a major role in determining the properties of the product for specific end uses. However, there are no reports in the literature that the authors are aware of to understand the evolution of microstructure in 3D printed HDPE/UHMWPE blends with controlled process parameters. Moreover, a comparison of the microstructural evolution for HDPE/UHMWPE blends between the 3D printing process and other conventional processing techniques is essential to understand the advantages of 3D printing and to correct any shortcomings 3D printing has in addition to identifying the differences in microstructures formed from different PE blends versus its neat components. Some significant challenges in this field yet to be addressed are the limitations of materials that can be 3D printed and understanding microstructural evolution that occurs during the actual process of injection molding or 3D printing as opposed to current studies that look into the microstructure after the materials are converted to a final product or test strip. In injection molding, 3D printing, or other forms of extrusion, the dynamic evolution of the degree of crystallinity, their orientation, amorphous orientation, and free volume distribution in the materials as it changes through the process steps still remain unexplored. Without that, we may only have a partial grasp of controlling the final properties of the resulting product.

In the current work, the variation of texture of HDPE and trimodal reactor blends made of HDPE/UHMWPE/HDPE_wax fabricated through 3D printing and injection molding is studied through a detailed pole figure and analysis of the orientation distribution functions (ODF) with the help of texture components. The microstructures of trimodal reactor blends with different molecular weights (*M*_w_) made of HDPE, nanophase-separated and unentangled UHMWPE and HDPE_wax fabricated using different 3D printing parameters in FFF and injection molding (control for processing) have been compared to those of HDPE (control for material) to look at the effect of 3D printing parameters, *M*_w_, processing, and composition. UHMWPE has a very low melt flow index (MFI) and cannot be 3D printed directly, so HDPE_wax, a good lubricant, was added to facilitate the process. Injection molding has been used as a control to look at the effects of material processing.

## 2 Experimental Methods

### 2.1 Synthesis of PE Reactor Blends

**Figure 1:**
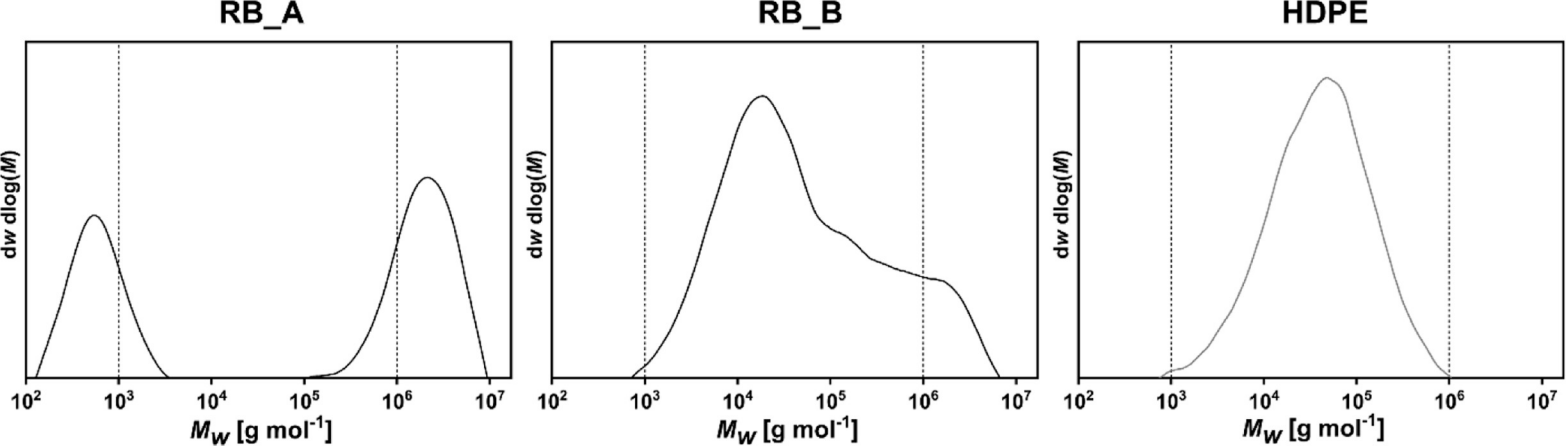
HT-SEC traces of the bimodal reactor PE blends (RB_A, base for RB1 and RB_B, base for RB2) and HDPE matrix and benchmark material.

The bimodal PE reactor blends containing UHMWPE (RB_A: 50 wt %; RB_B: 14 wt %; *M*_w_ > 10^6^ g mol^–1^), HDPE_wax (RB_A: 35 wt %; RB_B: <1 wt % Mw < 10^3^ g mol^–1^), and HDPE (RB_A: 15 wt %; RB_B: 86 wt %; 10^3^ g mol^–1^ < *M*_w_ < 10^6^ g mol^–1^) were tailored by ethylene polymerization on chromium-based single-site catalysts in collaboration with Mülhaupt lab in the Freiburg Materials Research Center and Institute for Macromolecular Chemistry at the University of Freiburg, Germany. The procedure has been published previously.^24,25^ HDPE (Hostalen GC7260; MFI = 8 g (10 min)^−1^), used as the matrix material and as the benchmark to investigate the influence of UHMWPE, was supplied by LyondellBasell. The high-temperature size exclusion chromatography (HT-SEC) traces of reactor blends RB_A, base for RB1, and RB_B, base for RB2, as well as the HDPE matrix and benchmark polymer investigated in the work, are shown in Figure 1. To produce the investigated trimodal HDPE/UHMWPE/HDPE_wax PE blends with 10 wt % UHMWPE each, the reactor blend powders with a bimodal PE molar mass distribution (RB_A or RB_B) were suspended in acetone and stabilized with Irganox 1010 and Irgafos P168 (0.5 wt %, 1:1). HDPE [80 wt % with respect to reactor blend RB_A to create RB1 (10 wt % UHMWPE); 70 wt % with respect to RB_B to create RB2 (10 wt % UHMWPE)] was added, and the solvent was evaporated under reduced pressure until a constant weight was reached. While significantly higher UHMWPE contents were demonstrated to be melt processable with extrusion and injection molding, the UHMWPE component in 3D printing had to be limited to 10 wt % as higher contents using the 3D printer model used for this research (see Section 2.2 for 3D printing model details) led to inconsistent 3D printing results most probably due to lack of proper mixing of components in the printing process. With ultrahigh-molecular-weight polymer chains, it takes additional time and additional process steps to mix them well when a higher percentage of UHMWPE is used. There may be another possibility that the UHMWPE could phase separate, with higher concentration, a possibility that was not investigated in this work as there are reports of phase separation in UHMWPE/liquid paraffin blend.^64^ To maximize the resulting properties from UHMWPE and to avoid any artifact in properties resulting from a nonuniform distribution of components in the mix, 10 wt % of UHMWPE has been used in this study.^24,25^ Melt processing was performed using the corotating twin-screw micro compounder Xplore from DSM at 200 °C and 120 rpm for 2 min. Afterward, the polymer melt was either injection molded (IM) to produce test specimens or extruded and granulated in preparation for filament extrusion for 3D printing.

### 2.2 Fabrication of IM and 3D Printed Samples

Injection molding has been used as the reference as it is a conventional processing technique. A DSM Xplore compounder combined with the DSM Xplore injection molding device was used for injection molding.^24,25,65^ Filaments for FFF were produced by using a TEACHLINE ZK 25 T twin-screw extruder from Collin at 25 rpm and 220 °C equipped with a 3.0 mm nozzle. The extruded polymer strands were pulled off (46–48 mm s^–1^), water-cooled (40 °C), and wound onto a spool. An Ultimaker 2+ FFF printer with the feed motor mounted directly onto the print head rather than on the back of the printer, as supplied, was used. For the trimodal reactor blends, the 3D printing parameters of printing speed and printing orientation were varied with a constant nozzle diameter of 0.8 mm and a temperature of 220 °C at which the optimum mechanics, warpage, molecular orientation, and processing capacity of the 3D printer were found for printing the samples. Similar conditions were used for 3D printing HDPE except for temperature, where 190 °C was found to give optimum conditions. Combinations of various samples with different printing speeds and printing orientations were investigated. Based on the measured polymer throughput at different 3D printing speeds, the related shear rate γ̇ and elongational strain rate ε̇ for the polymer melt were estimated according to eqs 1 and 2
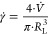
1
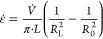
2where *V̇* is the polymer volume flow, measured for different 3D printing speeds, *L* = 2.4 mm is the length of the tapered area of the nozzle, *R*_L_ = 0.4 mm is the nozzle radius at the outlet, and *R*_0_ = 1.6 mm is the nozzle radius at the inlet. 3D printing at a printing speed of 25 mm s^–1^ led to γ̇ = 110 s^–1^ and ε̇ = 4.4 s^–1^ and a printing speed of 150 mm s^–1^ led to γ̇ = 450 s^–1^ and ε̇ = 18.3 s^–1^.

### 2.3 Crystallography

Samples of IM and 3D printed HDPE, RB1 and RB2 were cut into 1 cm × 1 cm × 0.2 cm dimensions and analyzed using an X-ray diffractometer (XRD), Philips X’Pert PW3050 materials research diffractometer XRD assembled with a pole figure goniometer, operating at 40 mA and 45 kV with Ni filtered Cu-K_α_ radiation. Wide angle X-ray diffraction (WAXD) was used to study the samples’ texture. A 1 mm × 1 mm collimator was used to produce a well-defined and focused beam.

A pole figure is defined as a stereographic projection, with a specified orientation relative to the specimen, indicating the pole density variation with pole orientation for a selected set of crystal planes.^66^ This selected set of crystal planes is chosen from the corresponding 2θ scan performed previously for a particular material. For orthorhombic PE, a minimum of five diffraction planes is needed to construct a pole figure.

The 2θ scan was performed with a step size of 0.0167° at a scan speed of 0.05°/s from 4 to 70°. A step size of 5° was used to generate the XRD data. The pole figures have been constructed using the MATLAB/MTEX software package designed to generate pole figures from XRD data. XRD equipment already accounts for defocusing, and the MTEX data analysis program corrects the texture for background data and accounts for sample misalignment and a drop in measured intensity near the edge of the sample due to geometric considerations to generate the pole figures. The values of lattice parameters according to the crystallographic information file used in interpreting the XRD data are *a* = 1.57 nm, *b* = 1.05 nm, and *c* = 0.75 nm, approximately.

The ODF of the samples’ crystallites gives more quantitative insights into the effect of texture. It is defined as the volume fraction of crystallites having a specific orientation (*g*) with respect to the sample coordinate system and it is the rotation required for the sample coordinate system [extension direction/rolling direction (ED/RD or *x*-axis), transverse direction (TD or *y*-axis) and normal direction (ND or *z*-axis)] to coincide with the crystal coordinate system.^8^ Bunge’s notation of ϕ_1_Φϕ_2_ has been used throughout this study. The sample coordinate system is first rotated about the *Z*-axis through ϕ_1_, then about the new *X*-axis through Φ and then about the *Z*-axis in the latest orientation through ϕ_2_ for Bunge’s notation.

## 3 Results and Discussion

### 3.1 Synthesis/Fabrication of IM and 3D Printed Samples

**Figure 2:**
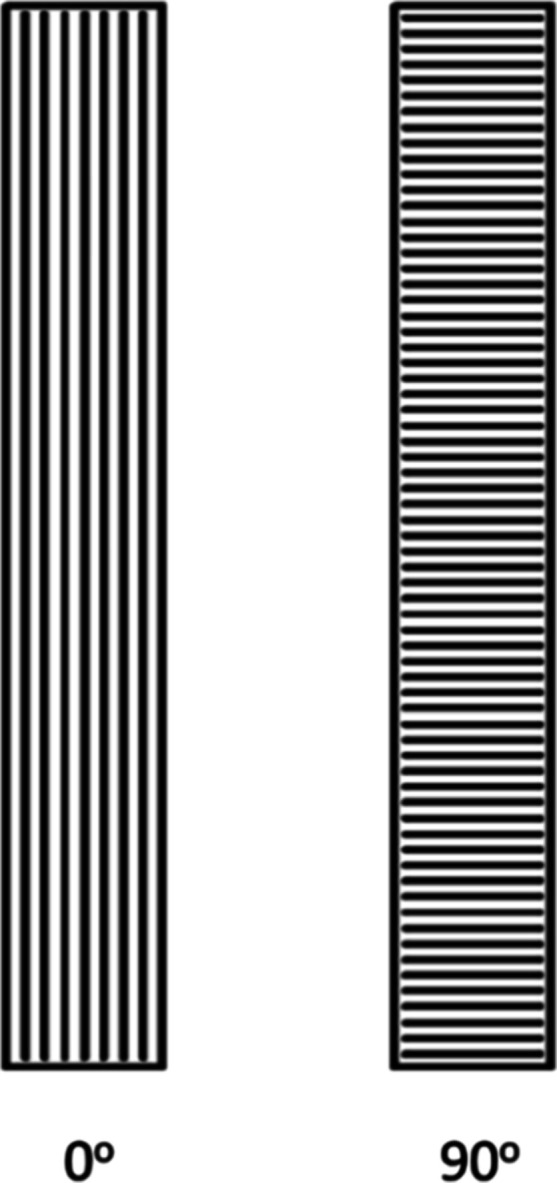
Printing orientations of 3D printed polymeric samples.

Table 1 shows the various processing conditions under which the samples have been obtained. Figure 2 shows the orientations, 0° and 90°, in which samples have been 3D printed, respectively. In other words, the parallel lines within the 3D printed samples in Figure 2 represent the 3D printing direction.

**Table 1:** Parameters for Samples Obtained by 3D Printing and Injection Molding

sample	process	nozzle diameter (mm)	printing orientation	printing speed (mm/s)
HDPE_IM	injection molding			
HDPE_0_25	FFF	0.8	0°	25
HDPE_0_150	FFF	0.8	0°	150
HDPE_90_25	FFF	0.8	90°	25
RB1_IM	injection molding			
RB1_0_25	FFF	0.8	0°	25
RB1_0_150	FFF	0.8	0°	150
RB1_90_25	FFF	0.8	90°	25
RB2_IM	injection molding			
RB2_0_25	FFF	0.8	0°	25
RB2_0_150	FFF	0.8	0°	150
RB2_90_25	FFF	0.8	90°	25

### 3.2 Texture and Crystallography

**Figure 3:**
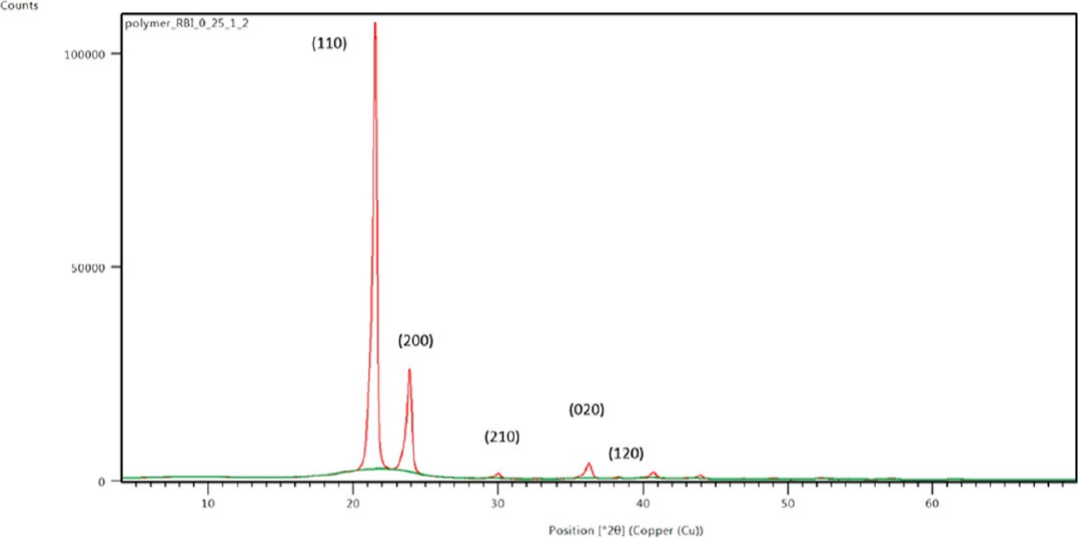
Representative 2θ scan of HDPE/UHMWPE samples.

A representative 2θ scan of the HDPE/UHMWPE samples is shown in Figure 3. The five peaks with the highest intensities obtained from the 2θ scan for the orthorhombic PE used in this work are (110), (200), (210), (020), and (120) corresponding to 2θ angles of 21.483, 23.849, 29.951, 36.146, and 39.205° respectively.

**Figure 4:**
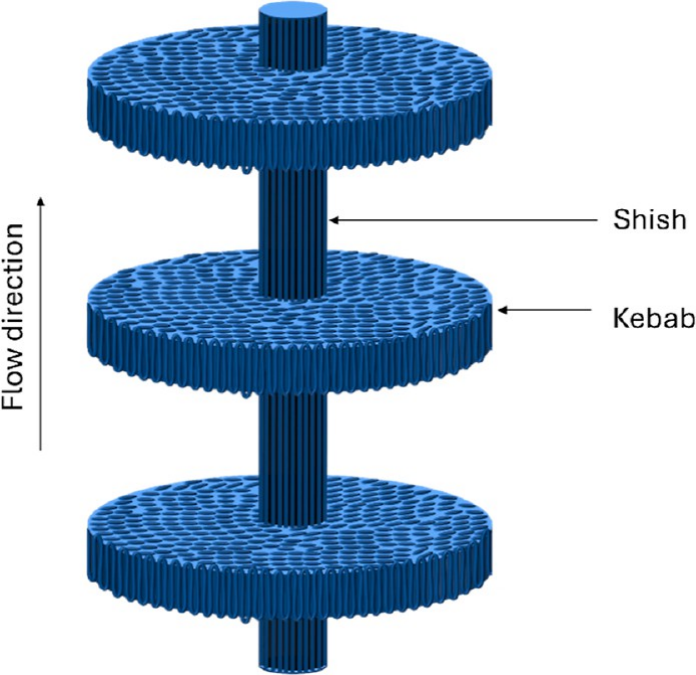
Schematic illustration of a shish kebab structure in UHMWPE present samples.

**Figure 5:**
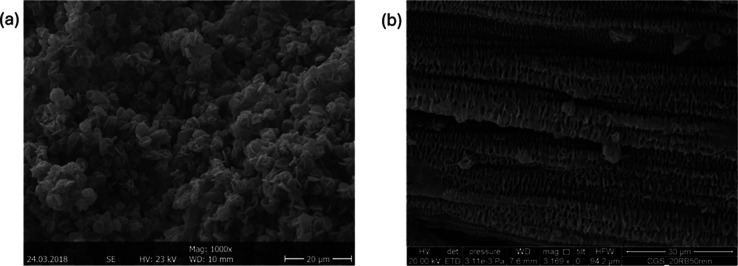
(a) SEM image of HDPE_0_150. (b) SEM image of RB1_0_150.

In correlation with the existing literature mentioned in the introduction, during the processing of UHMWPE, RB1, and RB2 samples consisting of UHMWPE, they were found to have shish kebab structures, while HDPE alone did not possess any such structures. Figure 4 shows a schematic illustration of an ideal shish kebab structure. The kebab part can be of different sizes. Figure 5a shows scanning electron microscopy (SEM) image of HDPE_0_150 showing no shish kebab structures, while Figure 5b shows the SEM image of RB1_0_150 showing the presence of shish kebab-type structures.

#### 3.2.1 Pole Figures of IM and 3D Printed Samples

**Figure 6:**
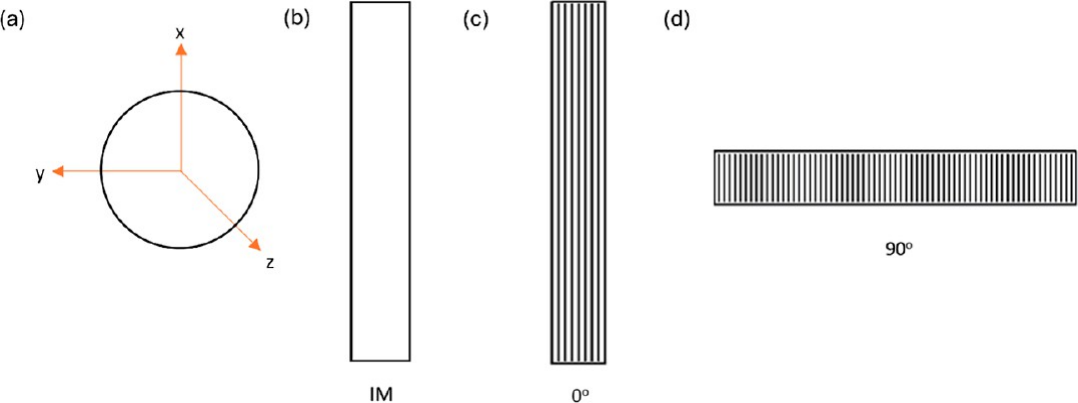
Schematic illustration of orientation of *x*, *y*, and *z* axes in (a) pole figure, (b) IM sample, (c) 3D printed (0° printing direction) sample, and (d) 3D printed (90° printing direction) sample [please note that the directions of *x*, *y*, and *z* axes shown in Figure 3.3 (a) apply to the rest of Figures 3.3(b), 3.3(c), and 3.3(d) too.].

**Figure 7:**
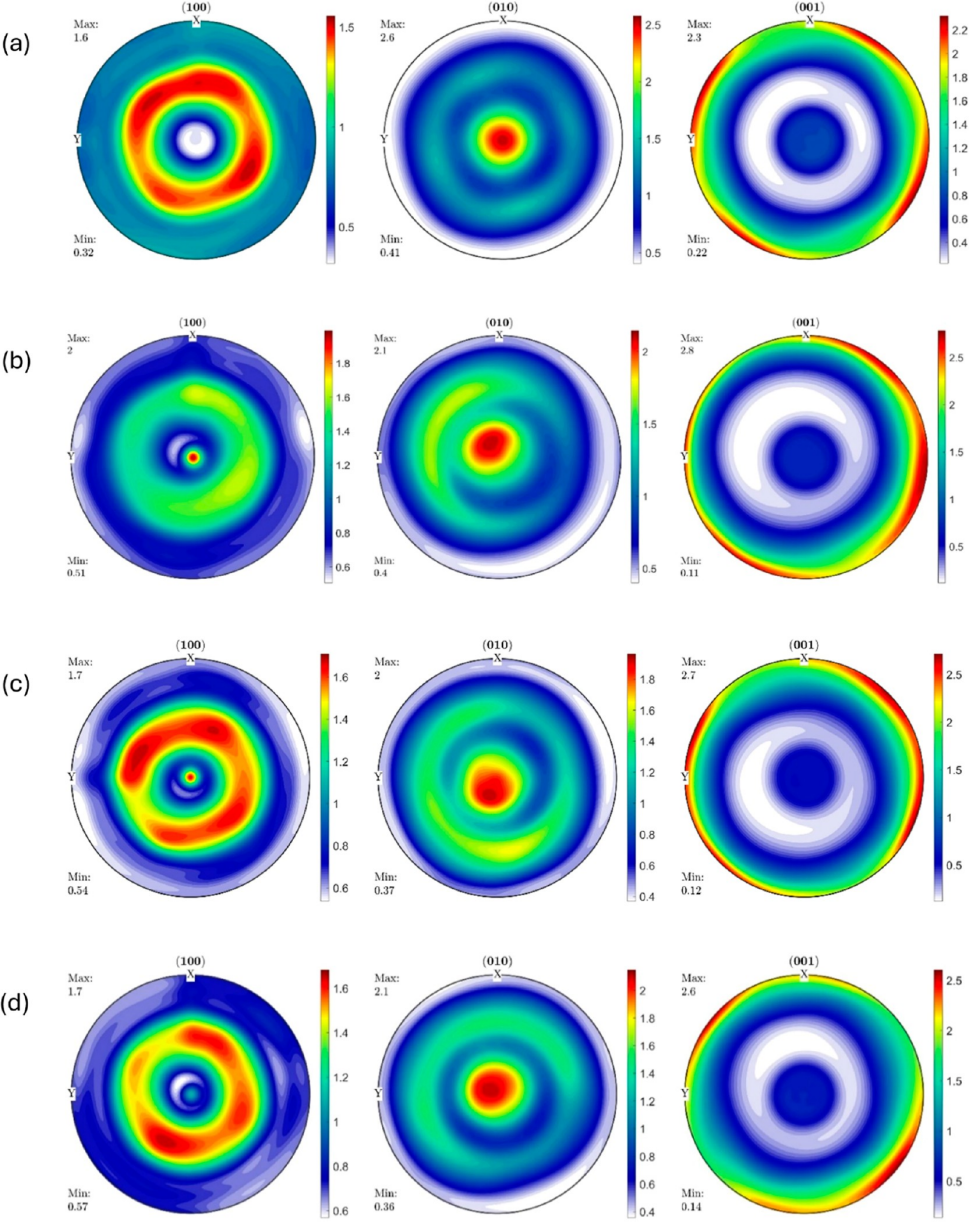
Pole figures of (a) HDPE_IM, (b) HDPE_0_25, (c) HDPE_90_25, and (d) HDPE_0_150.

**Figure 8:**
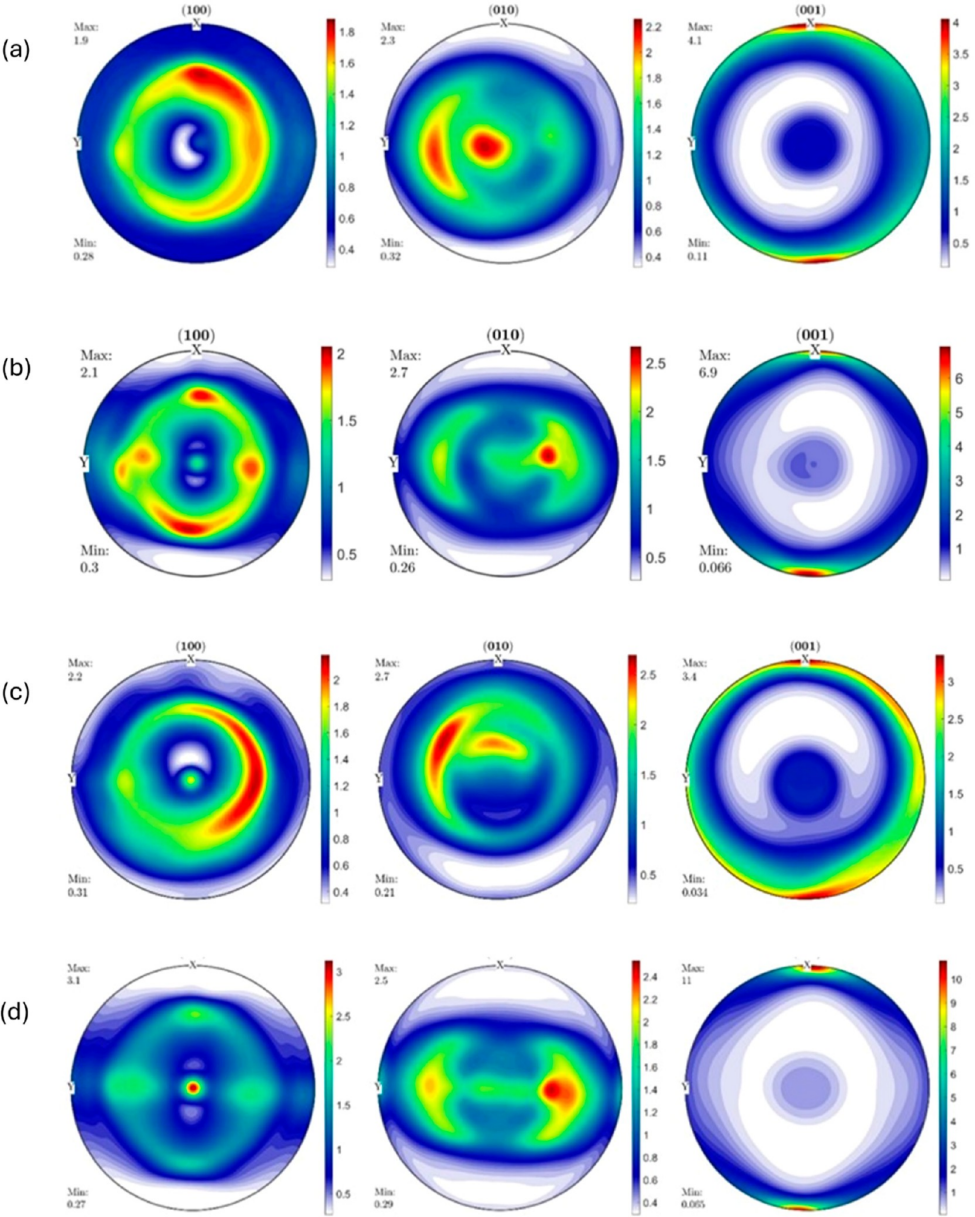
Pole figures of (a) RB1_IM, (b) RB1_0_25, (c) RB1_90_25, and (d) RB1_0_150.

**Figure 9:**
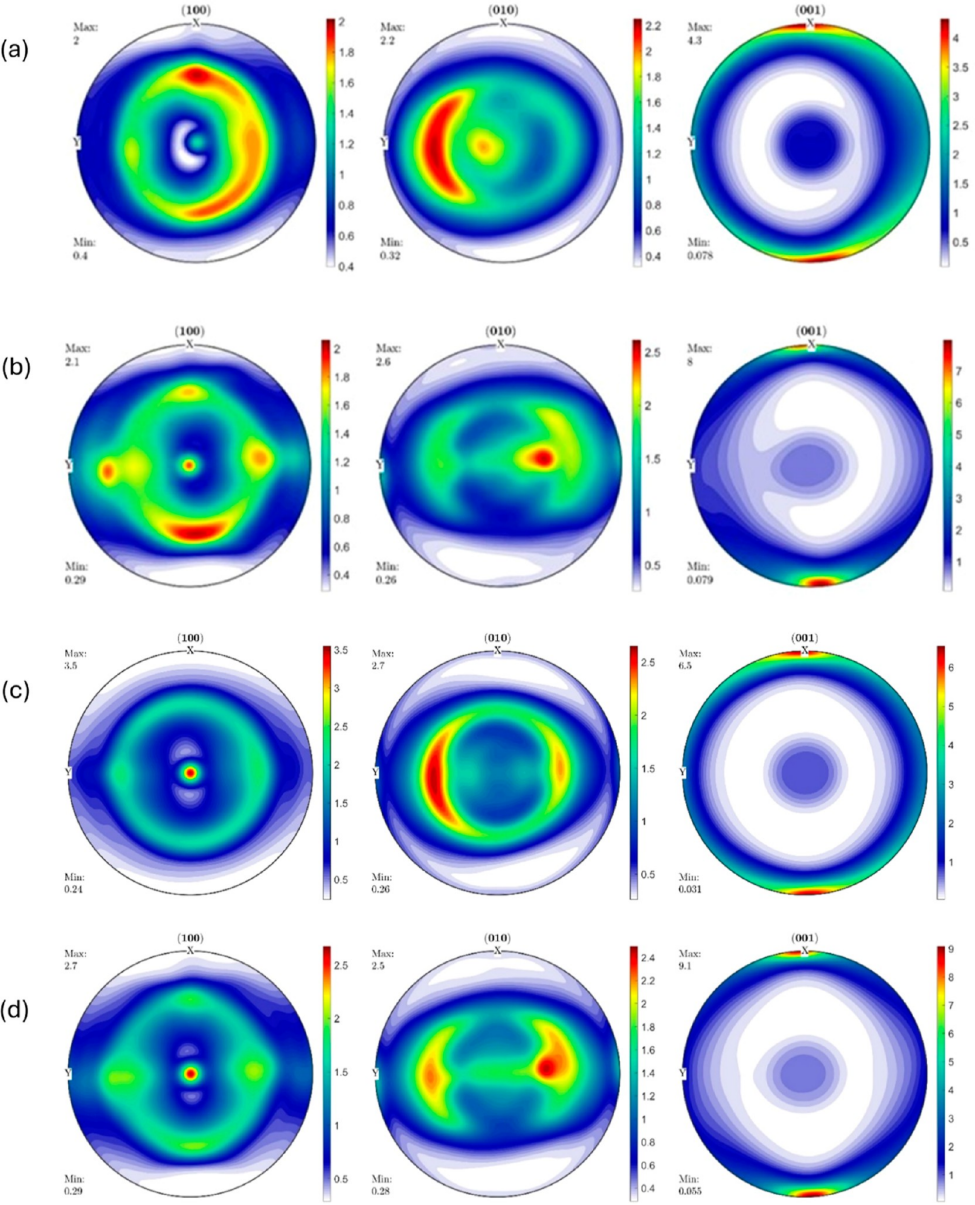
Pole figures of (a) RB2_IM, (b) RB2_0_25, (c) RB2_90_25, and (d) RB2_0_150.

The outward normal to the pole figure at its center represents the ND|*z*-axis of the corresponding sample, while the ED/RD|*x*-axis and TD|*y*-axis are represented by the vertical and horizontal directions, respectively. A schematic showing *x*, *y*, and *z* axes in all pole figures, IM, and 3D printed (both 0 and 90° directions) samples has been shown in Figure 6. Please note that the directions of *x*, *y*, and *z* axes shown in Figure 6a apply to the rest of Figure 6b–d too. The parallel lines within the 3D printed samples in the illustration represent the 3D printing direction, while the major axes of the figures represent the crystalline orientation. The ED/RD axis, i.e., *x*-axis, is along the printing direction for 3D printed samples and along the length of the samples (predominantly in the flow direction) for IM ones, while the TD axis, i.e., *y*-axis, is transverse/perpendicular to the printing direction for 3D printed samples and perpendicular to the length of the samples for IM ones. The ND axis, i.e., *z*-axis, is along the outward normal to the plane at the center of the samples. Each pole figure has a logarithmic scale of intensity with a varying range, with say, 10 representing intensity that is ten times the intensity of a randomly distributed microstructure, or in other words, the intensity represents the preferred orientation of the crystal planes or the probability/probability density of finding the corresponding crystal planes oriented in that direction. The pole figures of all the IM and 3D printed samples are shown in Figures 7–9.

The IM HDPE sample (HDPE_IM) shows almost a fibrous texture with a very low overall maximum intensity of 2.6, as seen on the (010) pole figure. The [010] crystal planes are clustered around the ND axis and axisymmetric about the ND axis. The [001] crystal planes with a maximum intensity of 2.3, as seen in the (001) pole figure, are clustered at the circumference of the ED–TD plane at about 45 and −45° to the TD–ND plane in both directions. In the (100) pole figure, the maximum intensity has a reduced value of 1.6, and it is observed that the [100] crystal planes lie axisymmetrically about the ND axis in the ED–TD plane.

The HDPE sample (HDPE_0_25) 3D printed at 25 mm s^–1^ at 0° orientation shows a significant difference in the (100) pole figure compared to that of HDPE_IM. The maximum texture intensity, seen on the (001) pole figure, has a slight increase to 2.8. In this specimen, the [001] crystal planes are clustered around the circumference of the ED–TD plane. The maximum intensity with a reduced value of just two on the (100) pole figure is seen to be clustered around the ND axis or the (001) specimen axis. In this sample, the maximum intensity of 2.1 in the (010) pole figure is seen to be clustered in the ED–TD plane around the ND axis. All three pole Figures (100), (010), and (001) pole figures are axisymmetric about the ND axis with low intensities. The pole figures indicate that the HDPE_0_25 sample has a good fiber texture, although with low intensities.

The HDPE sample (HDPE_90_25) 3D printed at 25 mm s^–1^ at 90° orientation shows a slight difference in the (100) pole figure in terms of intensity distribution and almost no difference in the (010) and (001) pole figures compared to those of HDPE_0_25. There is not much difference in the maximum texture intensities exhibited on all the pole figures compared to the HDPE_0_25 sample. All three (100), (010), and (001) pole figures are almost axisymmetric about the ND axis with similar intensities compared to HDPE_0_25 samples. Once again, the pole figures indicate that the HDPE_90_25 sample, although with low intensity, does have some fiber texture prevalence with a slightly changed preferential orientation distribution of [100] crystal planes as seen from (100) and (001) pole figures due to the change in 3D printing orientation.

The HDPE sample (HDPE_0_150) 3D printed at 150 mm s^–1^, i.e., higher shear and elongational strain rate, at 0° orientation shows a considerable difference in the (100) pole figure compared to that of HDPE_0_25, a slight difference in the (001) pole figure, and almost no difference in the (010) pole figure compared to those of HDPE_0_25. The overall maximum intensity is about the same in both HDPE_0_150 and HDPE_0_25 and is still found in the (001) pole figure. All three pole figures indicate that HDPE_0_150 also shows fiber texture prevalence with low intensities.

The IM RB1 sample (RB1_IM) shows a maximum texture intensity of 4.1 on the (001) pole figure, which is slightly higher than that in HDPE_IM. The (001) pole figure corresponding to the [001] direction shows that the [001] crystal planes have a preferred orientation near the ED, and the pole figure is axisymmetric about the ED–ND plane. The (010) pole figure shows a maximum intensity of 2.3° along the TD–ND plane. The maximum texture intensity on the (100) pole figure is seen to be extended until a rotation of about 45° from the TD–ND plane. The pole figure is almost axisymmetric around the ND axis, with a maximum intensity of 1.9. This observation indicates that a semifiber texture developed in the RB1 sample during injection molding. Also, this suggests that the addition of UHMWPE shish kebab structures changed the preferred orientation of the crystalline components, especially the [001] crystal planes, which are clustered at the (100) poles different from that from HDPE_IM.

The RB1 sample (RB1_0_25) 3D printed at 25 mm s^–1^ at 0° orientation with a 0.8 mm nozzle shows a significant texture evolution compared to that of the RB1_IM and HDPE_0_25. The maximum texture intensity on the (001) pole figure increased to 6.9. Here also, the (001) pole figure shows that the [001] crystal planes have a preferred orientation near the ED and are clustered at the (100) poles of the specimen. The maximum intensity of 2.1 on the (100) pole figure is seen only lying in the specimen’s *x*-axis (100) or the *y*-axis (010) family. The (010) pole figure shows a maximum intensity of 2.7 along the TD-ND plane at only one region making the probability of finding [010] planes lying along the TD–ND plane less. The (001) pole figure is axisymmetric about the ED–ND plane with an increased intensity. The (100) pole figure is axisymmetric about the ND axis, with the (010) pole figure being almost axisymmetric around the ND axis. This observation indicates that a significant semifiber texture is developed in the sample through 3D printing compared to injection molding of RB1_IM.

The RB1 sample (RB1_90_25) 3D printed at 25 mm s^–1^ at 90° orientation shows far less texture evolution compared to that of RB1_0_25. The maximum texture intensity, seen on the (001) pole figure, is only 3.4, which is almost half that of RB1_0_25. The (001) pole figure shows that [001] crystal planes have a preferred orientation near the ED and are clustered at (100) poles of the specimen, similar to RB1_0_25 and RB1_IM. The (010) pole figure shows a similar maximum intensity of 2.7 compared to that of the (010) pole figure of RB1_0_25, but the maximum probability of finding [010] planes is rotated off the TD-ND plane by about a few degrees, possibly due to the change in printing orientation. The (100) pole figure has a maximum intensity of 2.2 and is axisymmetric about the ED–ND plane, with the [100] planes having a preferred orientation below the TD–ND plane. The (001) pole figure has maximum intensity distributed symmetrically about the ED–ND plane and the ND axis. This indicates the presence of a semifiber texture similar to RB1_IM and RB1_0_25.

The RB1 sample (RB1_0_150) 3D printed at 150 mm s^–1^ at 0° orientation shows a remarkable difference in (100) and (001) pole figures compared to those of RB1_0_25 with a very slight change in the (010) pole figure. The maximum texture intensity on the (001) pole figure increased from 6.9 to 11 compared to 4.1 in the IM sample. In this specimen as well, the (001) pole figure shows that the [001] crystal planes exhibit a preferred orientation near the ED and are clustered at the (100) poles of the specimen. The maximum intensity on the (100) pole figure is now predominantly clustered around the ND axis or the (001) axis with a slightly increased intensity of 3.1 from 2.1. Once again, the (010) pole figure shows a maximum intensity of 2.5 along the TD–ND plane at only two regions, with a slightly increased intensity on one side with respect to the ND axis, along the TD–ND plane. The (001) pole figure is axisymmetric about the ED–ND plane with an increased intensity. The (100) pole figure is axisymmetric about the ND axis with slightly increased intensity as well, which indicates that the prevalent semifiber texture has notably evolved compared to the RB1_0_25 specimen.

The IM RB2 sample (RB2_IM) shows similar pole figure profiles compared to RB1_IM with almost no difference in maximum intensities of the (100), (010), and (001) pole figures. The overall maximum texture intensity of 4.3 is found in the (001) pole figure in this case, where the [001] crystal planes are clustered around the (100) specimen poles and are axisymmetric about the ED–ND plane. The (010) pole figure is axisymmetric about the TD–ND plane, and the maximum intensity of 2.2 is clustered around the TD–ND plane. The maximum intensity on the (100) pole figure is just two, and the [100] crystal planes have a preferred orientation on one side of the ED–ND plane, with the probability of finding the [100] planes lying on the ED–TD plane axisymmetric about the ND axis. Once again, it is observed that a semifiber texture is developed in the RB2 sample with a low intensity.

The RB2 sample (RB2_0_25) 3D printed at 25 mm s^–1^ at 0° orientation shows a noteworthy texture evolution compared to RB2_IM and has similar pole figure profiles compared to those of RB1_0_25. The maximum intensity is similar in (100) and (010) pole figures compared to those of RB1_0_25, while it slightly increased from 6.9 to 8 for the (001) pole figure. The [001] crystal planes have a preferred orientation near the ED axis at the (100) poles of the specimen. The maximum intensity of 2.1 on the (100) pole figure is seen in the ED–ND plane or TD–ND plane, i.e., in the specimen’s (100) or (010) family. The [010] planes have a preferred orientation at only one region along the TD–ND plane and the (010) pole figure is axisymmetric about the TD–ND plane with a maximum intensity of 2.6. This suggests significant semifiber evolution compared to the low intensity semifiber texture in the IM samples.

The RB2 sample (RB2_90_25) 3D printed at 25 mm s^–1^ at 90° orientation shows a remarkable difference in texture evolution compared to RB2_0_25 as well as RB1_90_25. The maximum texture intensity is 6.5 on the (001) pole figure, which is a slight decrease from 8 on the RB2_0_25 but a considerable increase from 3.4 on the RB1_90_25. The [001] crystal planes have a preferred orientation near the ED-axis poles similar to RB1_90_25 and RB2_0_25 but with an increased intensity, implying that the [001] crystal planes have a greater preferred orientation along the ED-axis. The (001) pole figure is axisymmetric about the ED–ND plane. The (010) pole figure shows a similar maximum intensity of 2.7 compared to both RB2_0_25 and RB1_90_25. However, the maximum texture intensity distribution differs slightly from that of RB2_0_25. The [100] crystal planes in the (100) pole figure show a maximum intensity of 3.5 with a slightly changed intensity distribution and are clustered around the ND axis. All the pole figures are almost axisymmetric about the ND axis, showing a semifiber to fiber texture prevalence.

The RB2 sample (RB2_0_150) 3D printed at 150 mm/s at 0° orientation shows a maximum intensity of 9.1, which is a slight increase from 8 in RB2_0_25 but a decrease from 11 in the RB1_0_150. All of the pole figure profiles are similar to that in RB1_0_150. The (001) pole figure is axisymmetric about the ND axis and shows that the [001] crystal planes have a preferred orientation near the ED axis and are clustered at the (100) poles of the specimen. The [010] planes are clustered around the TD–ND plane with a maximum intensity of 2.5, lying on one side of the ED–ND plane. The (010) pole figure is also axisymmetric about the ND axis. The (100) pole figure shows a maximum intensity of 2.7 with [100] planes clustered around the ND axis and axisymmetrical about the ND axis. All the pole figures are axisymmetric about the ND axis, suggesting again a semifiber to fiber texture prevalence in RB1_0_150.

The slight changes in intensities and their distributions whenever there is a change in printing speed or printing orientation can be attributed to various factors, including printing speed-dependent shear and elongational strain rates during the 3D printing process, cooling rate change of the material, and the path change of the nozzle during 3D printing. The differences in RB2 and RB1 when there is no change in 3D printing parameters can be attributed to the increased overall molecular weight of RB2 compared to RB1.

#### 3.2.2 ODFs and Their Schematics Showing Texture Components of IM and 3D Printed Samples

By convention, the three rotation parameters ϕ_1_Φϕ_2_ in Bunge’s notation are represented by Cartesian coordinates in a three-dimensional (3D) Euler space. The ODFs have a logarithmic scale of intensity with a varying range with, say, 12 representing an intensity that is 12 times the intensity of a randomly distributed microstructure.

**Figure 10:**
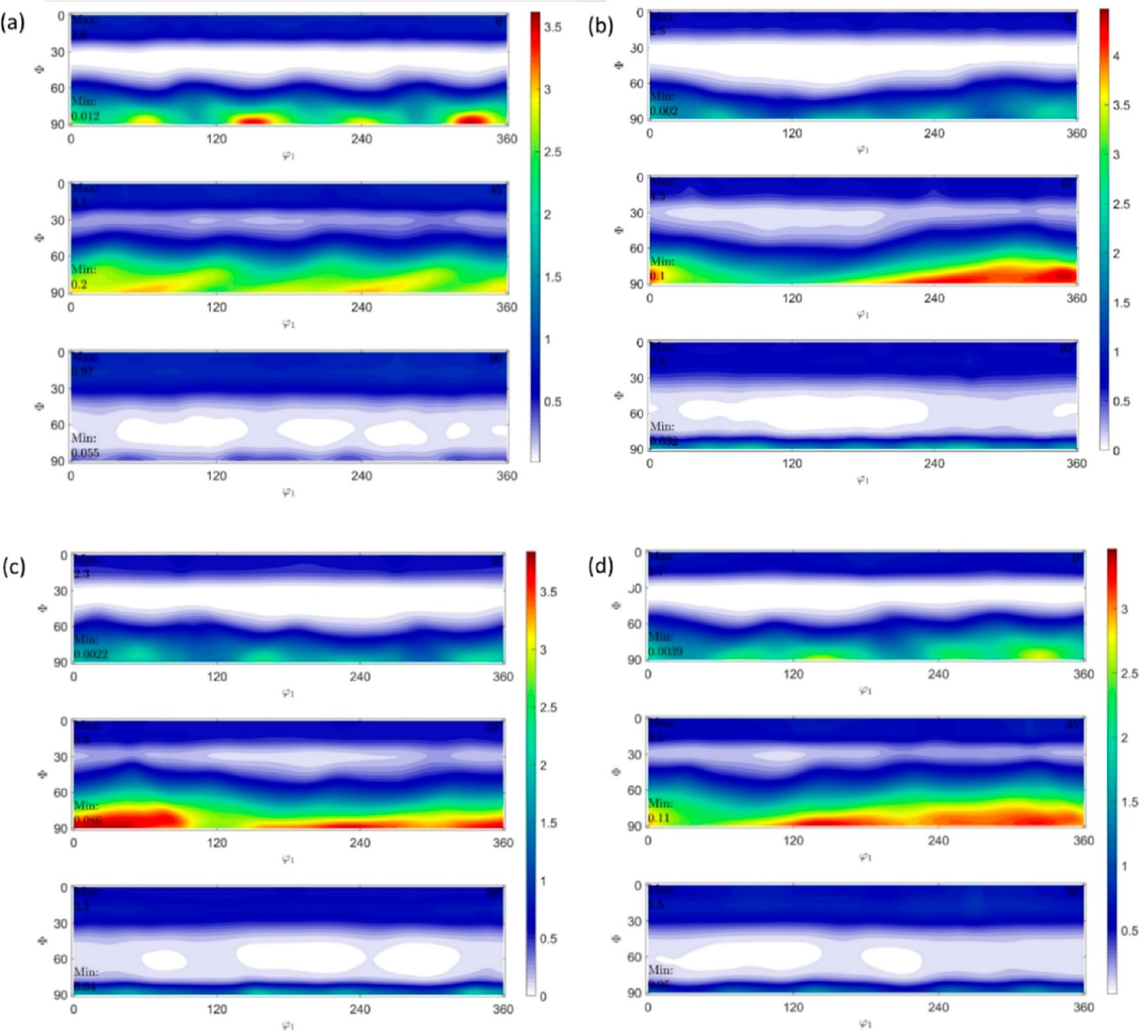
ODFs of (a) HDPE_IM, (b) HDPE_0_25, (c) HDPE_90_25, and (d) HDPE_0_150 at ϕ_2_ = 0, 45, and 90°.

**Figure 11:**
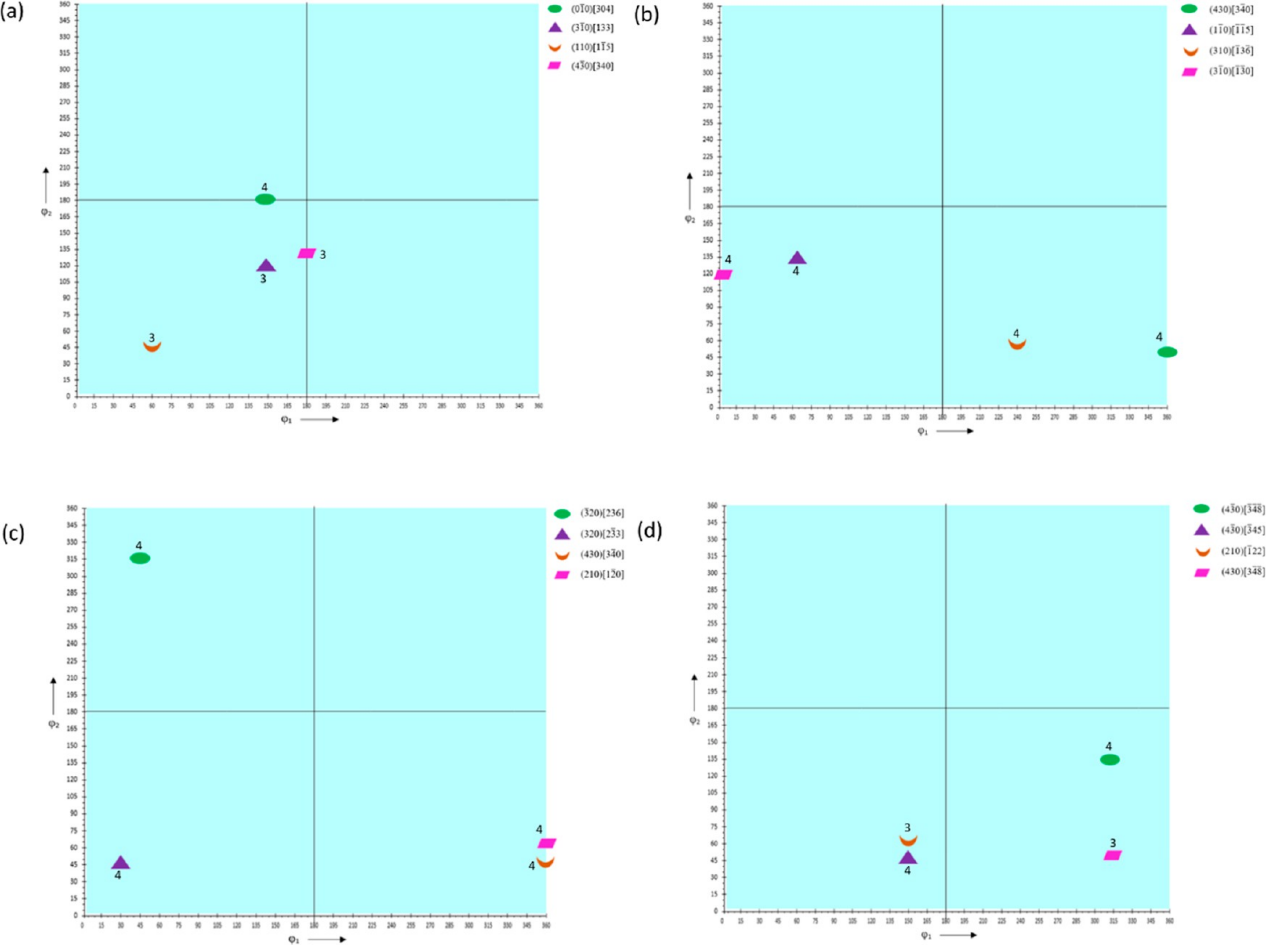
ODF schematics of (a) HDPE_IM, (b) HDPE_0_25, (c) HDPE_90_25, and (d) HDPE_0_150 at Φ = 90°.

**Figure 12:**
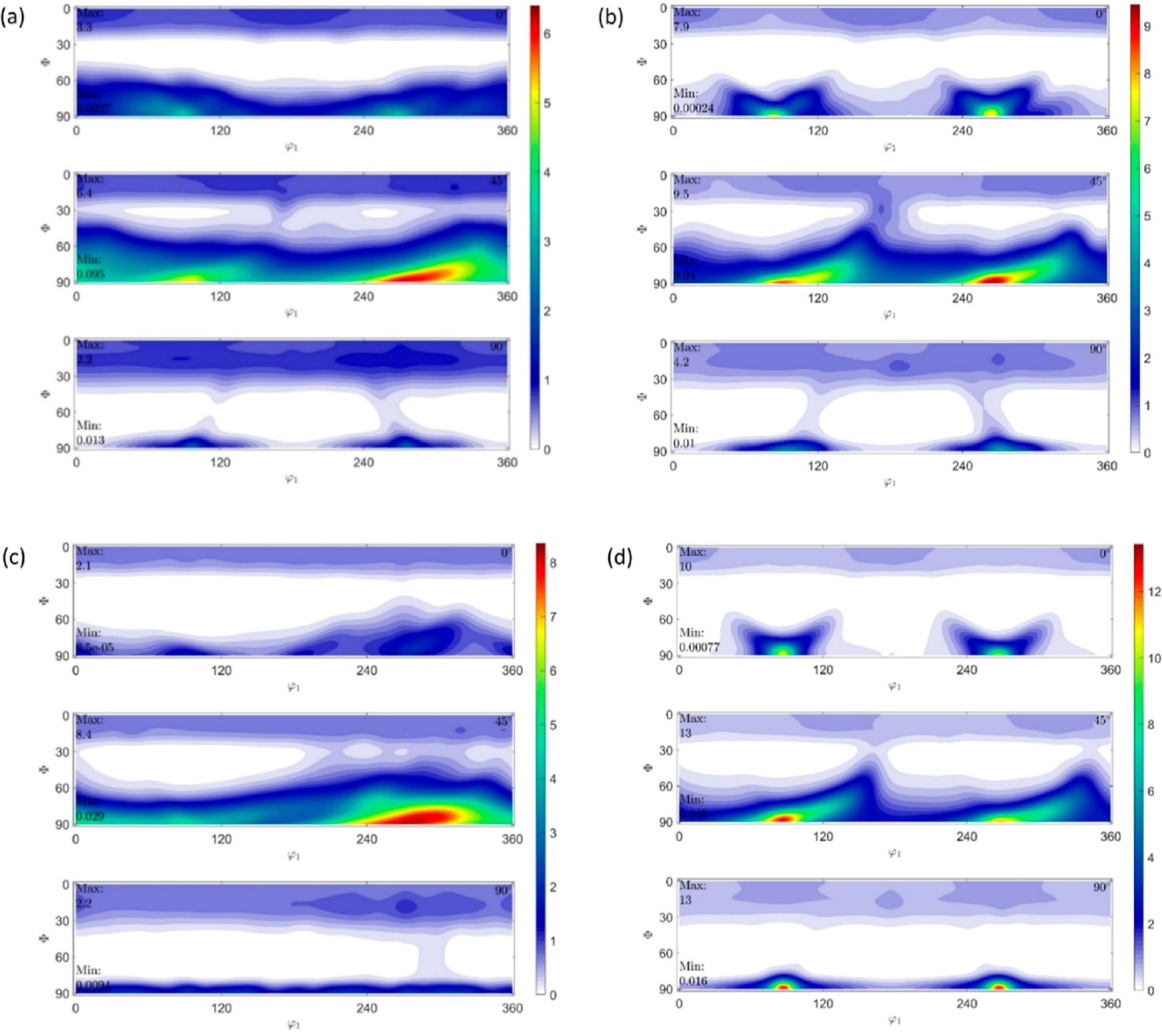
ODFs of (a) RB1_IM, (b) RB1_0_25, (c) RB1_90_25, and (d) RB1_0_150 at ϕ_2_ = 0, 45, and 90°.

**Figure 13:**
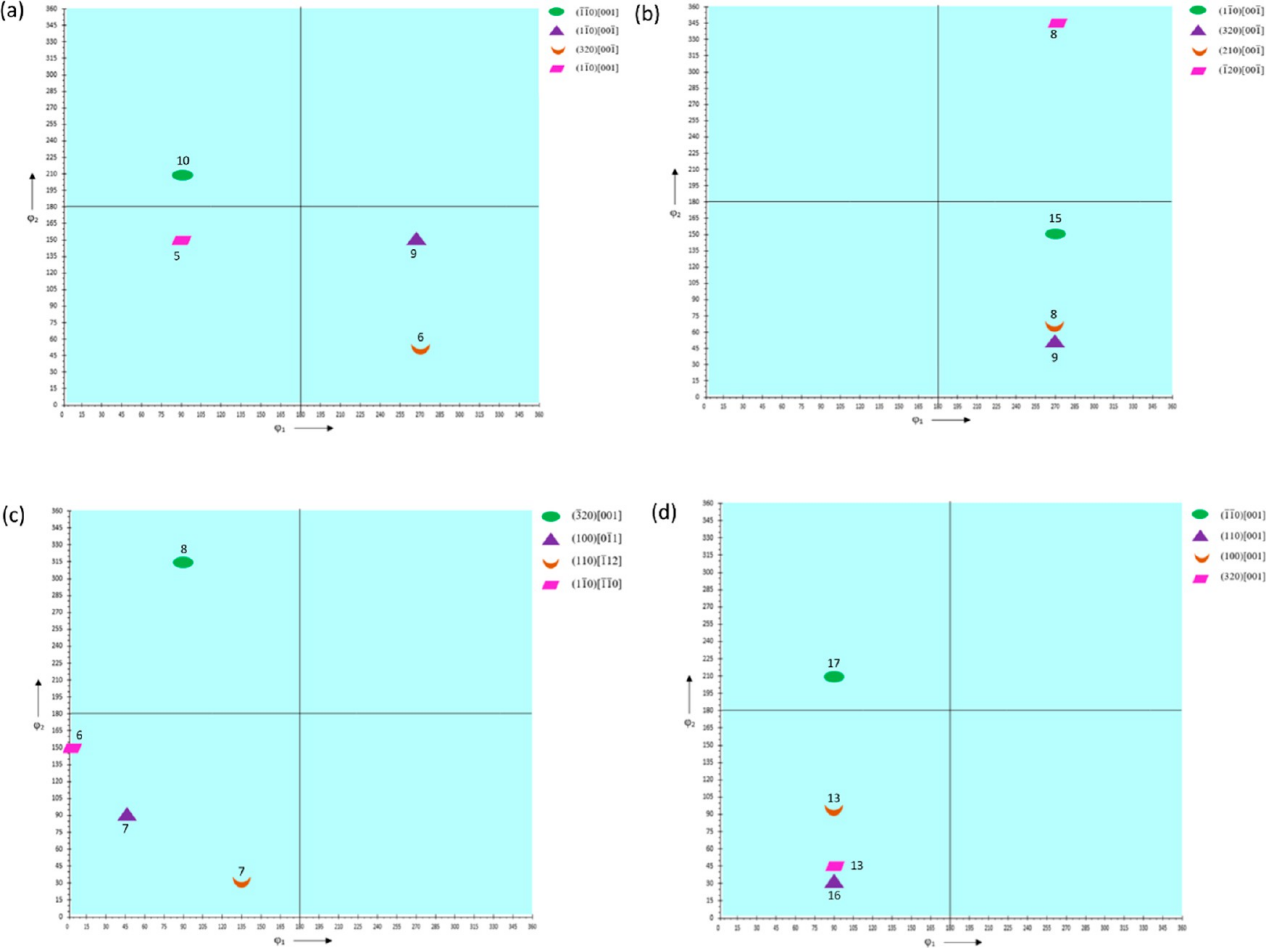
ODF schematics of (a) RB1_IM, (b) RB1_0_25, (c) RB1_90_25, and (d) RB1_0_150 at Φ = 90°.

**Figure 14:**
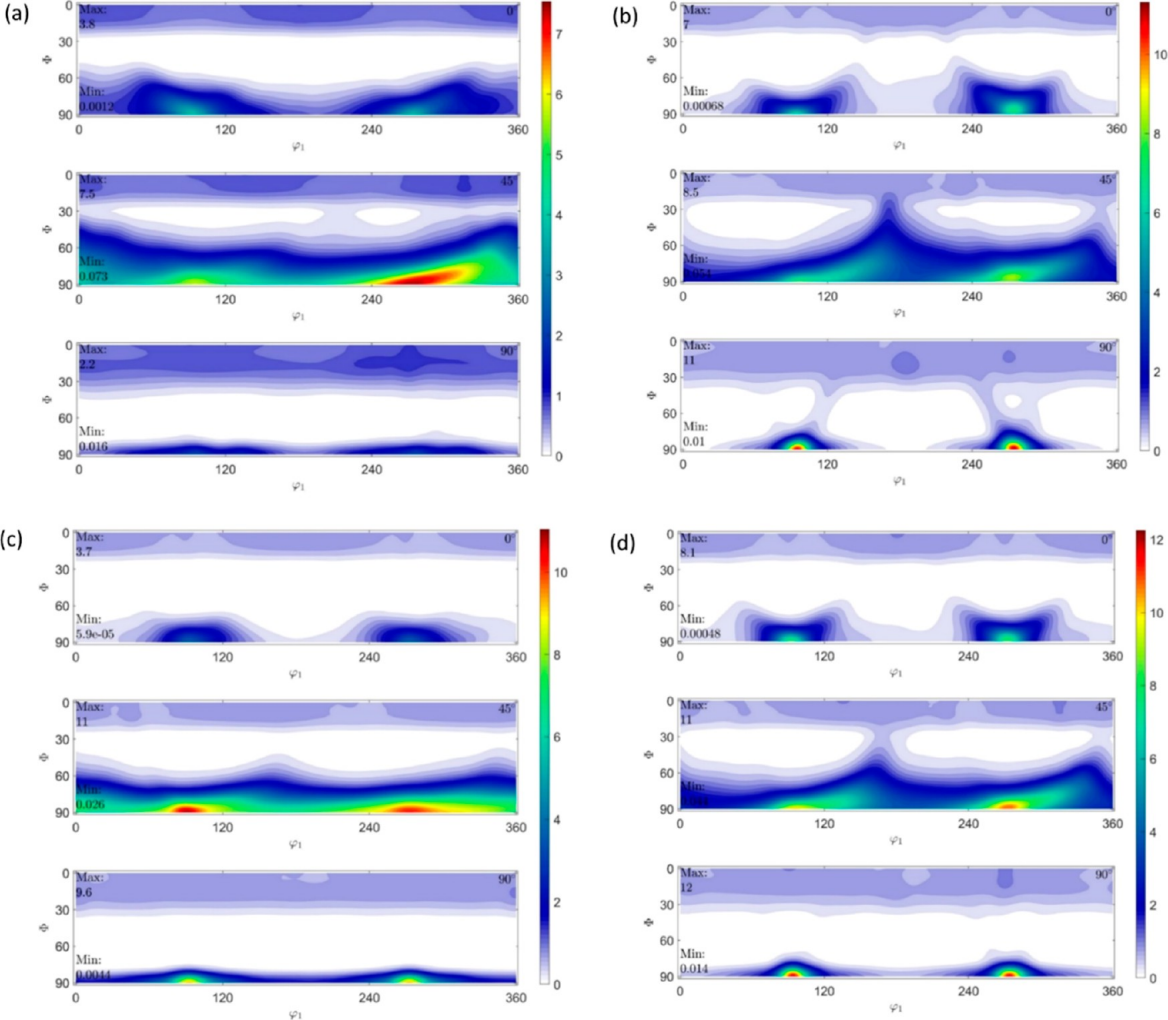
ODFs of (a) RB2_IM, (b) RB2_0_25, (c) RB2_90_25, and (d) RB2_0_150 at ϕ_2_ = 0, 45, and 90°.

**Figure 15:**
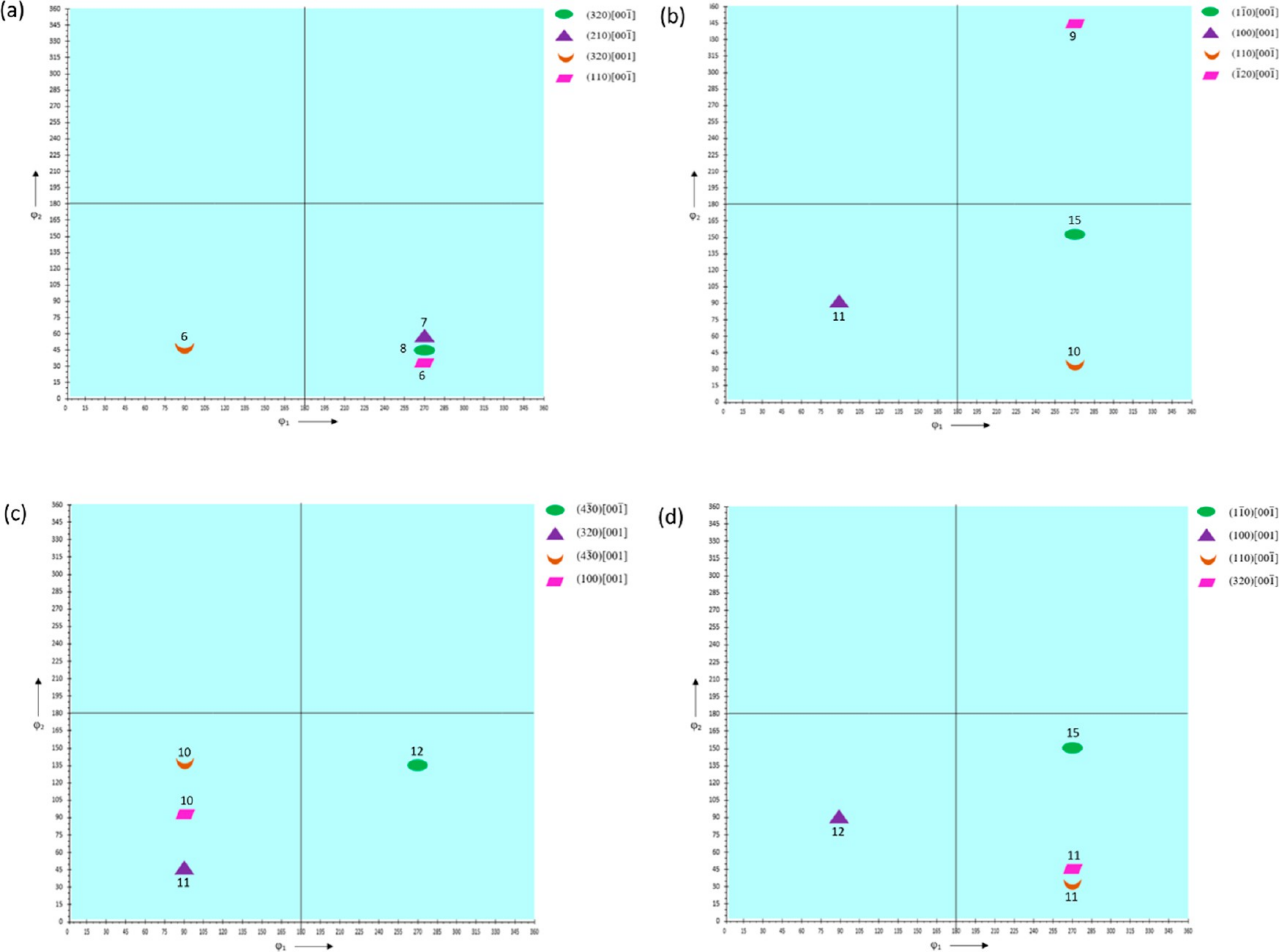
ODF schematics of (a) RB2_IM, (b) RB2_0_25, (c) RB2_90_25, and (d) RB2_0_150 at Φ = 90°.

The ODF plots constructed for ϕ_2_ = 0, 45, and 90° are shown in Figures 10, 12, and 14 for HDPE, RB1, and RB2, respectively. The intensities are obtained from the ODF plots and the extracted ODF data from MTEX/MATLAB. Although some components are rotated off the ϕ_1_, Φ, and ϕ_2_ axes by around 10–15°, the Miller indices can still be considered valid and are within accepted variance due to no definite grain boundaries present considering the existence of amorphous regions in the polymer samples. Due to the sample being polymeric having both crystalline and amorphous regions, the texture intensities have a spread of around 20–30° in general. The ODF schematics shown in Figures 11, 13, and 15 for HDPE, RB1, and RB2, respectively, depict the locations for the corrected Euler angles for the highest intensities of texture components. Also, only the components with the four highest texture intensities are represented in the ODF schematics in each case. The texture components with the highest six intensities for each of the specimens are given in the Supporting Information, along with their intensities rounded to the nearest integer and their corresponding Euler angles. The component with the highest experimental intensity is highlighted in green in each case in the Supporting Information to depict the true driving force within the texture. It must be noted that all of the major texture components lie in Φ = 90°. Hence, the ODF schematics of the samples are plotted at Φ = 90°.

In HDPE_IM, (01®0)[304] is the most prevalent texture component with an experimental intensity of 3.63, which is the highest for this sample, although it is relatively low compared to the very high intensities of other samples discussed further. In HDPE_0_25, the (430)[34®0] component has the highest intensity of 4.46, slightly higher than that of the IM HDPE_IM but comparatively lower than that of other high-intensity component samples. Also, most texture components are oriented in similar directions to those of HDPE_IM and have similar intensities, implying that the preferential orientation of crystal planes has only changed slightly due to 3D printing of HDPE at 25 mm/s and 0° printing orientation. In HDPE_90_25, the (3®20)[236] component has the highest experimental intensity of 3.94, which is similar to that in HDPE_IM and HDPE_0_25 and relatively low compared to other high-intensity samples where there is a greater preferential orientation of crystal planes. In HDPE_0_150, the highest intensity texture component is (43®0)[3®4®8®] with a slight decrease to 3.66. The low texture intensities indicate that not much texture is developed in 3D-printed HDPE with an increase in printing speed from 25 to 150 mm s^–1^.

In RB1_IM, the (1®1®0)[001] texture component has the highest intensity of 9.51. All the major components have the [001] or [001®], i.e., the *c*-axis of the orthorhombic crystallographic structure as the predominant direction, indicating that the planes oriented in the [001] and [001®] directions influence the mechanical response of the sample significantly. The increased intensities of texture components suggest that the addition of UHMWPE resulted in significant texture evolution with an increase in the preferential orientation of the crystal planes. (11®0)[001®] component has the highest experimental intensity of 15.18 in RB1_0_25. All of the intensities of major components are higher than those of injection-molded RB1_IM. However, all of the components are oriented in [001] or [001®] here. This observation suggests that 3D printing of RB1_0_25 at 25 mm s^–1^ and 0° printing orientation increases the preferential orientation of crystals compared to IM RB1_IM, with [001] and [001®] still existing as the predominant direction and remarkably contributing to the mechanical properties of the specimen. For RB1_90_25, (3®20)[001] is the component with the highest intensity of 8.42. The intensities of the first two major components showed a considerable decrease compared to those of RB1_IM and RB1_0_25, while other component intensities are comparable to those of RB1_IM but decreased compared to those of RB1_0_25. Only two of the major components lie in [001] or [001®] direction, suggesting that the preferential orientation has remarkably changed with the change in printing orientation from 0 to 90°. The (1®1®0)[001] texture component has the highest experimental intensity of 16.99 in RB1_0_150. Like RB1_IM and RB1_0_25, all the major components of RB1_0_150 are oriented in the [001] or [001®] direction. However, there is an increase in the intensities of the major components, suggesting an increase in the preferential orientation of crystal planes in the [001] and [001®] directions. Here, too, the planes oriented in the *c*-axis of the orthorhombic crystallographic structure influence the mechanical response of the specimen significantly.

The (320)[001®] texture component has the highest experimental intensity of 7.59 in RB2_IM, which is slightly lower than the highest experimental intensity of RB1_IM. The remaining major components have comparable intensities. Four major components orient in [001] or [001®] direction in this sample also and are similar to those of RB1_IM. Contrary to RB1_IM, two components are oriented in directions other than [001] or [001®]. This implies that composition change resulting in an overall higher molecular weight *M*_w_ has only influenced the texture considerably. In this specimen, too, the *c*-axis of the orthorhombic crystallographic structure is the predominant direction, and the planes oriented in [001] and [001®] substantially influence the mechanical properties of the sample. (11®0)[001®] texture component possesses the highest experimental intensity of 15.44 in RB2_0_25, which is very high compared to RB2_IM and similar to that of RB1_0_25. In this specimen, similar to RB1_0_25 and as opposed to RB2_IM, all the components are oriented in the [001] or [001®] direction, suggesting that 3D printing of RB2 at 25 mm s^–1^ and 0° printing orientation has resulted in texture evolution to a significant extent, making the planes oriented in the *c*-axis of the orthorhombic crystallographic structure contribute to the mechanical response of the sample. In RB2_90_25, (43®0)[001®] is the texture component with the highest experimental intensity of 12.00. This maximum intensity is higher than that of RB1_90_25 but lower than that of RB2_0_25. Importantly, all the components are oriented in [001] or [001®] direction with four similar to those in RB2_0_25, indicating that the preferential orientation of crystal planes still lies in [001] or [001®] direction even after the printing orientation changed. In RB2_0_150, the (11®0)[001®] texture component possesses the highest experimental intensity of 15.20, which is similar to the highest intensity of RB2_0_25. The remaining major components’ intensities increase slightly compared to RB2_0_25. All the components are oriented in the [001] or [001̅] direction and are similar to RB2_0_25. This suggests that an increase in printing speed from 25 to 150 mm s^–1^ resulted in only a very slight change in texture. In this sample, planes oriented in the [001] and [001̅] directions significantly influence the mechanical properties of the sample.

Almost similar intensities of the major components in all HDPE specimens, RB1_90_25, RB2_90_25, suggest that all of them contribute almost equivalently toward the mechanical properties including wear, hardness, modulus, etc. of the samples. For RB1_IM, RB1_0_25, RB1_0_150, RB2_IM, RB2_0_25, and RB2_0_150, planes oriented in [001] and [001®] directions significantly influence the mechanical properties of the samples.

#### 3.2.3 Discussion on the Mechanism of Fiber Texture Development

**Figure 16:**
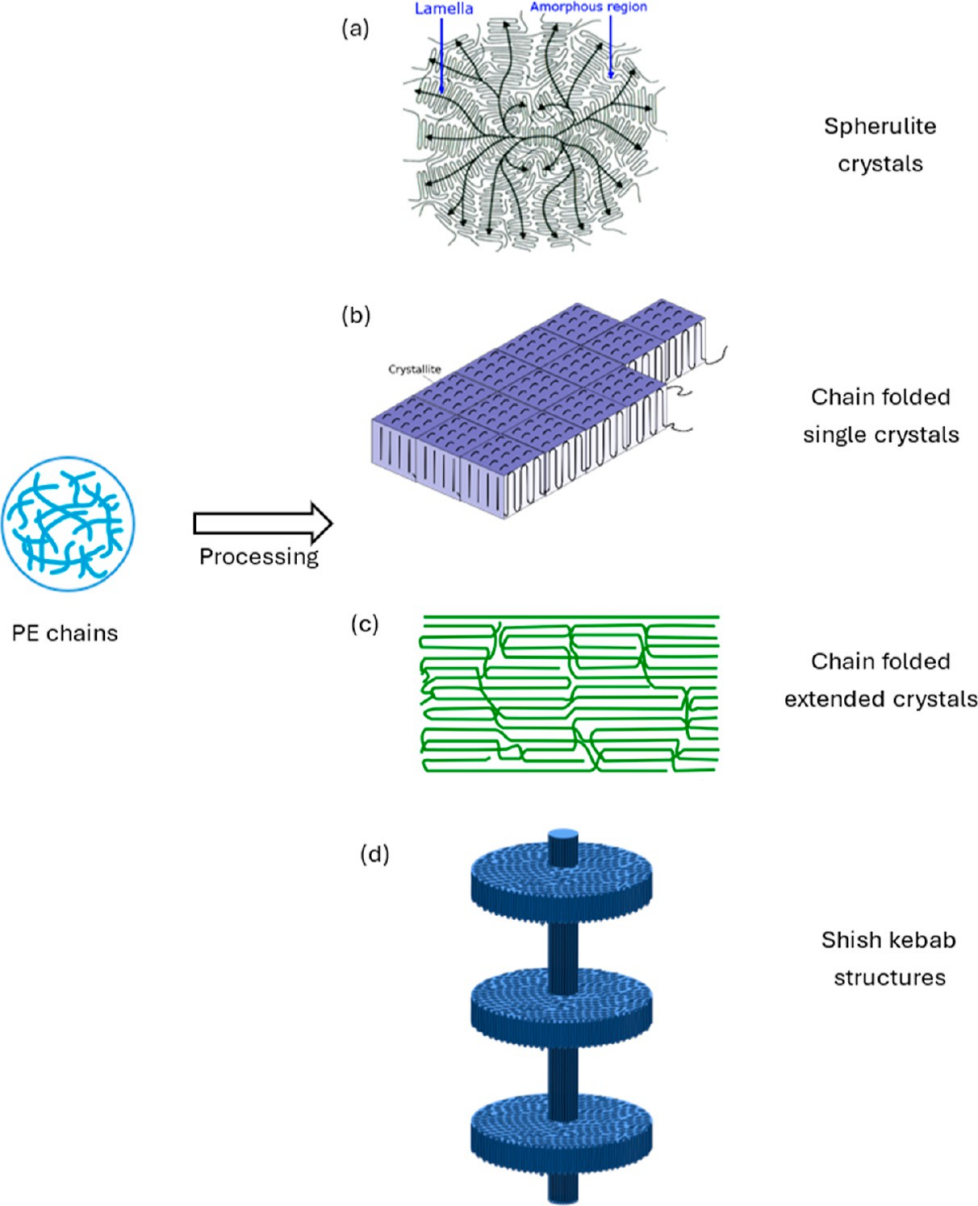
Schematic illustration of types of crystals formed that influence the fiber texture developed in HDPE, RB1, and RB2. (a) Spherulite crystals. (b) Chain-folded single crystals. (c) Chain-folded extended crystals. (d) Shish kebab structures.

Different types of crystals—chain folded single crystals, extended chain crystals, spherulite crystals, and shish-kebab crystal structures—all contribute to fiber texture development in RB1, RB2 blends, and HDPE. Pole figures and ODFs obtained from WAXD studies show the presence of low-intensity fiber-like texture in HDPE that develops into a high-intensity fiber texture (predominant unidirectional crystalline orientation) in RB1 and RB2 blends but does not give insights into mechanism of the fiber texture development. An online measuring technique that captures the texture development along the process line of 3D printing or injection molding (primarily synchrotron or advanced photon source) along with transmission electron microscopy techniques could help understand the mechanism of fiber texture development in the samples. The flow pattern and the viscosity evolution and the resulting stresses induced in the material during the processing affect the type and extent of crystallites formed, i.e., the mechanism and extent of fiber texture development. Figure 16 shows a schematic illustration of different types of crystals formed that influence the fiber texture developed in HDPE, RB1, and RB2 samples.

## 4 Conclusions

In this work, the texture evolution and change in the degree of anisotropy of 3D printed PE trimodal reactor blends and HDPE have been investigated and compared to those of the corresponding IM samples. Table 2 summarizes the highest-intensity texture components and their Euler angles. In HDPE, there is an evident fiber-like texture found in all samples, but there is little preferred orientation distribution of crystals as seen from the not-so-high intensities of the texture components. With the change in processing or 3D printing speed or printing orientation, there is some change in the preferred orientation of the crystals, i.e., there is a change in the major components but not their intensities in HDPE. The greater the intensity and texture component distribution in a sample, the higher the extent of anisotropy; i.e., greater intensity/component variation implies a higher degree of anisotropy induced in the sample, which affects wear and other anisotropic properties. With the presence of 10 wt % UHMWPE in RB1, the preferential orientation distribution of crystal planes changed significantly with relatively higher intensities, and the low-intensity fiber texture in HDPE changed to high-intensity semifiber to fiber texture in RB1. All the highest intensity major components in IM and 3D printed samples of RB1 printed in 0° orientation are observed to have crystals oriented in [001] or [001®]. In RB1, the change in processing from injection molding to 3D printing in a 0° orientation increased the intensities of the preferred orientation of the crystals. An increase in the printing speed further increased the intensities of the major texture components. Change in the printing orientation of RB1 shifted the preferential orientation of most of the major components with a decrease in their intensities compared to the IM RB1 and 3D printed RB1 at 0° orientation. An increase in the overall *M*_w_, while maintaining the UHMWPE content, of RB1 to give RB2 changed the preferential orientation distribution of the crystal planes to some extent, but the intensities of the texture components did not differ much when corresponding samples were compared to those of RB1 except for the 3D printed samples at 90° orientation. For 3D printed RB2 at a 90° orientation, the intensities are comparatively greater than those of 3D printed RB1 at 90° orientation and IM RB2 but lesser than 3D printed RB2 at 0° orientation. All the major components in IM and 3D printed samples of RB2 printed in both 0 and 90° orientations are observed to have crystals oriented in [001] or [001®]. The change in the processing of RB2 from injection molding to 3D printing increased the intensity of the preferred orientation of the crystals. The increase in printing speed and the change in printing orientation of RB2 changed the intensities and texture components to some extent. Overall, in both RB1 and RB2, the change in processing from injection molding to 3D printing at 0° orientation increased the intensities of the major components and increase in 3D printing speed, which is linked to an increase in shear and elongational strain rates, resulting in improved extension of the UHMWPE in the reactor blends, further increasing their intensities. In contrast, the change in 3D printing orientation decreased the intensities of the major texture components.

**Table 2:** Texture Components, Intensities, and Euler Angles of Highest Intensity Components for all Samples

sample	intensity	ϕ_1_ (deg)	Φ (deg)	ϕ_2_ (deg)	calculated (*hkl*)[*uvw*]	final (*hkl*)[uvw]
HDPE_IM	4	150	90	180	(01®0)[33 0 40]	(01®0)[304]
HDPE_0_25	4	360	90	45	(430)[27 4®0® 0]	(430)[34®0]
HDPE_90_25	4	45	90	315	(3®20)[236]	(3®20)[236]
HDPE_0_150	4	315	90	135	(43®0)	(43®0)[3®4®8®]
RB1_IM	10	90	90	210	(5®6®0)[001]	(1®1®0)[001]
RB1_0_25	15	270	90	150	(56®0)[001®]	(11®0)[001®]
RB1_90_25	8	90	90	315	(3®20)[001]	(3®20)[001]
RB1_0_150	17	90	90	210	(5®6®0)[001]	(1®1®0)[001]
RB2_IM	8	270	90	45	(320)[001®]	(320)[001®]
RB2_0_25	15	270	90	150	(55®0)[001®]	(11®0)[001®]
RB2_90_25	12	270	90	135	(43®0)[001®]	(43®0)[001®]
RB2_0_150	15	270	90	150	(56®0)[001®]	(11®0)[001®]

**Figure 17:**
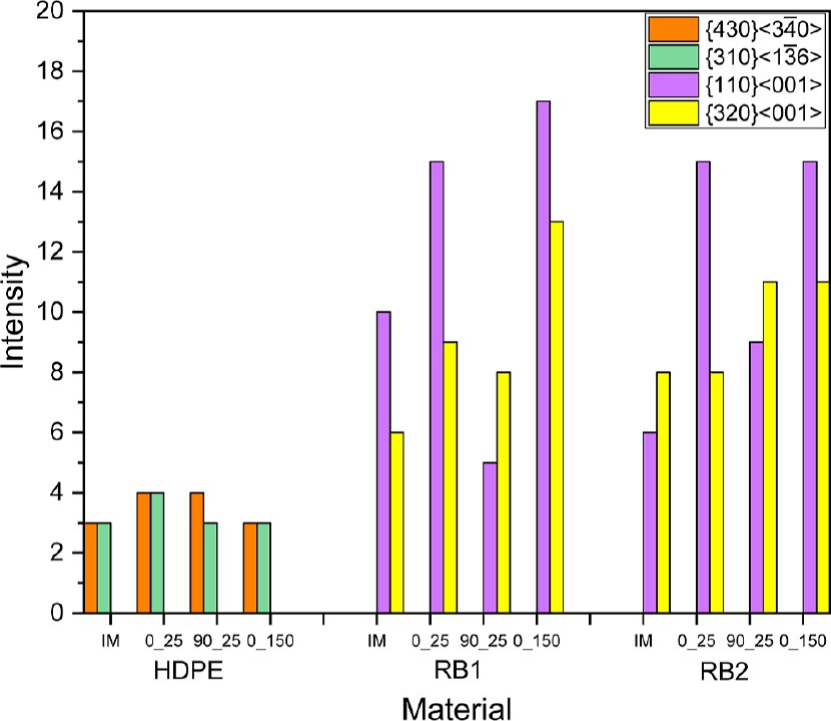
Variation of intensity of common texture components of IM and 3D printed samples.

Comparing the texture components of HDPE, RB1, and RB2, it is observed that {430}⟨34®0⟩ and {310}⟨13®6⟩ are common components for HDPE specimens while {110}⟨001⟩ and {320}⟨001⟩ are common in RB1 and RB2 specimens. It should be noted that (43®0)[001®] has been considered approximately as (11®0)[001®] while comparing common texture components. Figure 17 shows the common texture components’ intensity variation plotted for all the samples. For a family of crystal planes/orientations, the highest intensity is considered.

On the whole, changes in the preferred orientation distribution of crystals in 3D printed trimodal PE blends and HDPE were studied in comparison to IM trimodal PE blends and HDPE using WAXD through pole figures and ODFs. Parameters like overall *M*_w_ of the trimodal blend, 3D printing speed, and 3D printing orientation have been varied to investigate the variation in texture and texture components. It has been observed that the presence of UHMWPE (in trimodal blends versus HDPE) resulted in significant texture evolution of the samples in both 3D printed and IM samples. 3D printed trimodal PE blend samples in a 0° orientation showed a greater texture evolution and higher degree of anisotropy than corresponding IM samples. Also, an increase in 3D printing speed increased texture, while the change in 3D printing orientation decreased texture compared to IM samples. The *M*_w_ of the trimodal PE blends changed the preferred orientation distribution of the crystallites to some extent, but there has not been much change in the degree of anisotropy. It is observed that the process parameters (processing method, 3D printing speed, and 3D printing orientation) affect texture to a greater extent than material parameters (overall *M*_w_ of the blend).

## Supporting Information Available

Texture components, intensities, and Euler angles for all IM and 3D printed samples (PDF)
