# Supplementary material for: Crystallographic Texture Evolution in 3D Printed Polyethylene Reactor Blends
Source: ACS Omega. 2024 May 1;9(19):21016–34. doi: 10.1021/acsomega.4c00387 (PMC11097177; doi:10.1021/acsomega.4c00387)
Supplement: Supplementary file 1 — ao4c00387_si_001.pdf [file ao4c00387_si_001.pdf]

Crystallographic texture evolution in 3D printed polyethylene reactor blends

Sahitya Movva[1,5,*], Carl G. Schirmeister[2,3], Timo Hees[2], David Tavakoli[1], Erik H. Licht[3], Rolf Mülhaupt[2,4], Hamid Garmestani[1], Karl I. Jacob[1,6]

[1]*School of Materials Science and Engineering, Georgia Institute of Technology, Atlanta, GA 30332, USA*
[2] *Freiburg Materials Research Center FMF and Institute for Macromolecular Chemistry, Albert-Ludwigs-University Freiburg, Stefan-Meier-Str. 21, D-79104 Freiburg, Germany*
[3]*Basell Sales & Marketing B.V., LyondellBasell Industries, Industriepark Höchst, D-65926 Frankfurt a.M., Germany*
[4]*Sustainability Center Freiburg, Ecker-Str. 4, D-79104 Freiburg, Germany*
[5]*Intel Corporation, 2501 NE Century Blvd, Hillsboro, OR 97124, USA†*
[6]*G.W. Woodruff School of Mechanical Engineering, Georgia Institute of Technology, Atlanta, GA 30332, USA*

Abstract

In this work, crystallographic texture evolution in 3D printed trimodal polyethylene (PE) blends, and high-density polyethylene (HDPE) benchmark material were investigated to quantify the resulting material anisotropy, and the results were compared to materials made from conventional injection molded samples. Trimodal polyethylene reactor blends consisting of HDPE, ultra-high molecular weight polyethylene (UHMWPE), and HDPE_wax have been used for 3D printing and injection molding. Changes in the preferred orientation and distribution of crystallites, i.e., texture evolution, were quantified utilizing the Wide Angle X-ray diffraction (WAXD) through pole figures, and orientation distribution functions (ODFs) for 3D printed and injection molded samples. Since the change in weight-average molecular weight (M_w) of the blend was expected to significantly affect the resulting crystallinity and orientation, the overall M_w of the trimodal PE blend was varied while keeping the UHMWPE component weight fraction to 10% in the blend. The resulting texture was analyzed by varying the overall M_w of the trimodal blend and the process parameters in 3D printing and compared to the texture of conventional injection molded samples. The printing speed and orientation (defined with respect to the axis along the length of the samples) were used as the variable process parameters for 3D printing. The degree of anisotropy increases with an increase in the non-uniform distribution of intensities in pole figures and ODFs. All the highest intensity major texture components in injection molded and 3D printed samples (0° printing orientation) of reactor blends are observed to have crystals oriented in [001] or [$00\bar{1}$]. Overall, for the same throughput, 3D printed samples in the 0° orientation showed greater texture evolution and higher anisotropy compared to injection molded samples. Most notably, increase in 3D printing speed increased crystalline distribution closer to the 0° direction, increasing the anisotropy, while deviation from this printing orientation reduced crystalline distribution closer to the 0° direction, thus increasing isotropy. This demonstrates that tailoring material properties in specific directions can be achieved more effectively from 3D printing than from the injection molding process. Change in the overall M_w of the trimodal PE blend changed the preferential orientation distribution of the crystal planes to some degree. However, degree of anisotropy remained the same in almost all cases, indicating that the effect of molecular weight distribution is not as significant as the printing speed and printing orientation in tailoring the resulting

†This is only the author's current affiliation, and the current work has not been carried out at or in collaboration with Intel Corporation.

properties. The 3D printing process parameters (speed and orientation) were shown to have more influence on the texture than material parameters associated with the blend.

Keywords: Ultra high molecular weight polyethylene; High density polyethylene; 3D printing; Trimodal reactor blends; X-Ray Diffraction; Injection molding; Texture analysis; Pole figures; Orientation distribution functions.

**Corresponding author. Email address: sahitya.movva@intel.com ; sahitya6@gatech.edu*

Supporting Information

The supporting information for section '3.2.1. ODFs and their schematics showing texture components of IM and 3D printed samples' has been provided here.

The Miller indices that correspond to Euler angles with six highest intensities are calculated to identify the individual texture components from the ODF plots. The intensities, Euler angles and the corresponding Miller indices for the twelve samples are shown in Tables 2(a) to 2(l). Like mentioned in the main article, the intensities are obtained from both the ODF plots as well as the extracted ODF data from MTEX/MATLAB. The intensities are rounded to the nearest whole number and the highest experimental intensity is highlighted in green in the tables to depict the true driving force within the texture. Also, the texture components have been approximated for better comparison such that (hkl).[uvw] = 0 is satisfied. Both the calculated component values and final approximated component values with texture intensities are listed in Tables S1 to S12. Some components have been excluded due to crystal symmetry. It must be noted that all the major texture components lie in $\Phi = 90^o$.

**Intensity**	**φ_1 (º)**	**Φ (º)**	**φ_2 (º)**	**Calculated (hkl)[uvw]**	**Final (hkl)[uvw]**
4	150	90	180	$(0\bar{1}0)[33\ 0\ 40]$	$(0\bar{1}0)[304]$
3	150	90	120	$(14\ \bar{5}\ 0)[33\ 90\ 80]$	$(3\bar{1}0)[133]$
3	60	90	45	$(430)[2\ \bar{3}\ 10]$	$(110)[1\bar{1}5]$
3	180	90	135	$(4\bar{3}0)[27\ 40\ 0]$	$(4\bar{3}0)[340]$
3	60	90	0	(010)[207]	(010)[103]
3	60	90	60	$(310)[1\bar{3}6]$	$(310)[1\bar{3}6]$

Table S1: Texture components, intensities and Euler angles for HDPE_IM

**Intensity**	**φ_1 (º)**	**Φ (º)**	**φ_2 (º)**	**Calculated (hkl)[uvw]**	**Final (hkl)[uvw]**
4	360	90	45	$(430)[27\ \overline{40}\ \ 0]$	$(430)[3\bar{4}0]$
4	60	90	135	$(4\bar{3}0)[\bar{2}\ \bar{3}\ 10]$	$(1\bar{1}0)[\bar{1}\bar{1}5]$
4	240	90	60	$(310)[\bar{1}3\bar{6}]$	$(310)[\bar{1}3\bar{6}]$
4	0	90	120	$(3\bar{1}0)[\bar{1}\bar{3}0]$	$(3\bar{1}0)[\bar{1}\bar{3}0]$
3	330	90	60	$(14\ 50)[33\ \overline{90}\ \overline{80}]$	$(310)[1\bar{3}\bar{3}]$
3	135	90	30	$(890)[\bar{1}12]$	$(110)[\bar{1}12]$

Table S2: Texture components, intensities and Euler angles for HDPE_0_25

**Intensity**	**φ_1 (º)**	**Φ (º)**	**φ_2 (º)**	**Calculated (hkl)[uvw]**	**Final (hkl)[uvw]**
4	45	90	315	$(\bar{3}20)[236]$	$(\bar{3}20)[236]$
4	30	90	45	$(320)[2\bar{3}3]$	$(320)[2\bar{3}3]$
4	360	90	45	$(430)[27\ \overline{40}\ \ 0]$	$(430)[3\bar{4}0]$
4	360	90	60	$(520)[1\bar{2}0]$	$(210)[1\bar{2}0]$
3	225	90	30	$(890)[\bar{1}1\bar{2}]$	$(110)[\bar{1}1\bar{2}]$
3	60	90	120	$(3\bar{1}0)[\bar{1}\bar{3}6]$	$(3\bar{1}0)[\bar{1}\bar{3}6]$

Table S3: Texture components, intensities and Euler angles for HDPE_90_25

**Intensity**	**φ_1 (º)**	**Φ (º)**	**φ_2 (º)**	**Calculated (hkl)[uvw]**	**Final (hkl)[uvw]**
4	315	90	135	$(4\bar{3}0)[\overline{16}\ \overline{25}\ \overline{47}]$	$(4\bar{3}0)[\bar{3}\bar{4}\bar{8}]$
4	150	90	45	$(430)[\bar{6}\ 9\ 10]$	$(430)[\bar{3}45]$
3	150	90	60	$(520)[\bar{1}22]$	$(210)[\bar{1}22]$
3	315	90	45	$(430)[16\ \overline{25}\ \overline{47}]$	$(430)[3\bar{4}\bar{8}]$
3	0	90	135	$(4\bar{3}0)[\overline{27}\ \overline{40}\ \ 0]$	$(4\bar{3}0)[\bar{3}\bar{4}0]$
3	60	90	120	$(3\bar{1}0)[\bar{1}\bar{3}6]$	$(3\bar{1}0)[\bar{1}\bar{3}6]$

Table S4: Texture components, intensities and Euler angles for HDPE_0_150

Intensity	φ_1 (º)	Φ (º)	φ_2 (º)	Calculated (hkl)[uvw]	Final (hkl)[uvw]
10	90	90	210	$(\bar{5}60)[001]$	$(\bar{1}\bar{1}0)[001]$
9	270	90	150	$(5\bar{6}0)[00\bar{1}]$	$(1\bar{1}0)[00\bar{1}]$
6	270	90	45	$(320)[00\bar{1}]$	$(320)[00\bar{1}]$
5	90	90	150	$(5\bar{6}0)[001]$	$(1\bar{1}0)[001]$
5	270	90	315	$(\bar{3}20)[00\bar{1}]$	$(\bar{3}20)[00\bar{1}]$
5	90	90	120	$(3\bar{1}0)[001]$	$(3\bar{1}0)[001]$

Table S5: Texture components, intensities and Euler angles for RB1_IM

Intensity	φ_1 (º)	Φ (º)	φ_2 (º)	Calculated (hkl)[uvw]	Final (hkl)[uvw]
15	270	90	150	$(5\bar{6}0)[00\bar{1}]$	$(1\bar{1}0)[00\bar{1}]$
9	270	90	45	$(320)[00\bar{1}]$	$(320)[00\bar{1}]$
8	270	90	60	$(520)[00\bar{1}]$	$(210)[00\bar{1}]$
8	270	90	345	$(\bar{2}50)[00\bar{1}]$	$(\bar{1}20)[00\bar{1}]$
8	90	90	0	$(010)[001]$	$(010)[001]$
6	90	90	345	$(\bar{2}50)[001]$	$(\bar{1}20)[001]$

Table S6: Texture components, intensities and Euler angles for RB1_0_25

Intensity	φ_1 (º)	Φ (º)	φ_2 (º)	Calculated (hkl)[uvw]	Final (hkl)[uvw]
8	90	90	315	$(\bar{3}20)[001]$	$(\bar{3}20)[001]$
7	45	90	90	$(100)[0\bar{1}1]$	$(100)[0\bar{1}1]$
7	135	90	30	$(890)[\bar{1}12]$	$(110)[\bar{1}12]$
6	0	90	150	$(5\bar{6}0)[\bar{1}\bar{1}0]$	$(1\bar{1}0)[\bar{1}\bar{1}0]$
6	180	90	60	$(520)[\bar{1}20]$	$(210)[\bar{1}20]$
5	270	90	135	$(4\bar{3}0)[00\bar{1}]$	$(4\bar{3}0)[00\bar{1}]$

Table S7: Texture components, intensities and Euler angles for RB1_90_25

Intensity	φ_1 (º)	Φ (º)	φ_2 (º)	Calculated (hkl)[uvw]	Final (hkl)[uvw]
17	90	90	210	$(\bar{5}\bar{6}0)[001]$	$(\bar{1}\bar{1}0)[001]$
16	90	90	30	(890)[001]	(110)[001]
13	90	90	90	(100)[001]	(100)[001]
13	90	90	45	(320)[001]	(320)[001]
12	270	90	45	$(320)[00\bar{1}]$	$(320)[00\bar{1}]$
12	90	90	150	$(5\bar{6}0)[001]$	$(1\bar{1}0)[001]$

Table S8: Texture components, intensities and Euler angles for RB1_0_150

Intensity	φ_1 (º)	Φ (º)	φ_2 (º)	Calculated (hkl)[uvw]	Final (hkl)[uvw]
8	270	90	45	$(320)[00\bar{1}]$	$(320)[00\bar{1}]$
7	270	90	60	$(520)[00\bar{1}]$	$(210)[00\bar{1}]$
6	90	90	45	(320)[001]	(320)[001]
6	270	90	30	$(890)[00\bar{1}]$	$(110)[00\bar{1}]$
5	60	90	150	$(5\bar{6}0)[\bar{1}\bar{1}4]$	$(1\bar{1}0)[\bar{1}\bar{1}4]$
5	315	90	150	$(5\bar{6}0)[\bar{1}\bar{1}\bar{2}]$	$(1\bar{1}0)[\bar{1}\bar{1}\bar{2}]$

Table S9: Texture components, intensities and Euler angles for RB2_IM

Intensity	φ_1 (º)	Φ (º)	φ_2 (º)	Calculated (hkl)[uvw]	Final (hkl)[uvw]
15	270	90	150	$(5\bar{6}0)[00\bar{1}]$	$(1\bar{1}0)[00\bar{1}]$
11	90	90	90	(100)[001]	(100)[001]
10	270	90	30	$(890)[00\bar{1}]$	$(110)[00\bar{1}]$
9	270	90	345	$(\bar{2}50)[00\bar{1}]$	$(\bar{1}20)[00\bar{1}]$
8	270	90	45	$(320)[00\bar{1}]$	$(320)[00\bar{1}]$
7	270	90	120	$(3\bar{1}0)[001]$	$(3\bar{1}0)[001]$

Table S10: Texture components, intensities and Euler angles for RB2_0_25

Intensity	φ_1 (º)	Φ (º)	φ_2 (º)	Calculated (hkl)[uvw]	Final (hkl)[uvw]
12	270	90	135	$(4\bar{3}0)[00\bar{1}]$	$(4\bar{3}0)[00\bar{1}]$
11	90	90	45	(320)[001]	(320)[001]
10	90	90	135	$(4\bar{3}0)[001]$	$(4\bar{3}0)[001]$
10	90	90	90	(100)[001]	(100)[001]
9	90	90	30	(890)[001]	(110)[001]
8	270	90	30	$(890)[00\bar{1}]$	$(110)[00\bar{1}]$

Table S11: Texture components, intensities and Euler angles for RB2_90_25

Intensity	φ_1 (º)	Φ (º)	φ_2 (º)	Calculated (hkl)[uvw]	Final (hkl)[uvw]
15	270	90	150	$(5\bar{6}0)[00\bar{1}]$	$(1\bar{1}0)[00\bar{1}]$
12	90	90	90	(100)[001]	(100)[001]
11	270	90	30	$(890)[00\bar{1}]$	$(110)[00\bar{1}]$
11	270	90	45	$(320)[00\bar{1}]$	$(320)[00\bar{1}]$
10	90	90	45	(320)[001]	(320)[001]
9	270	90	345	$(\bar{2}50)[00\bar{1}]$	$(\bar{1}20)[00\bar{1}]$

Table S12: Texture components, intensities and Euler angles for RB2_0_150
